# The m6A reader YTHDC2 promotes the pathophysiology of temporal lobe epilepsy by modulating SLC7A11-dependent glutamate dysregulation in astrocytes

**DOI:** 10.7150/thno.100703

**Published:** 2024-09-03

**Authors:** Kai Zhang, Zhiquan Yang, Zhuanyi Yang, Liangchao Du, Yu Zhou, Shiyu Fu, Xiaoyue Wang, Xing Li, Dingyang Liu, Xinghui He

**Affiliations:** 1 Department of Neurosurgery, Xiangya Hospital, Central South University, Changsha, China.; 2 National Clinical Research Center for Geriatric Disorders, Xiangya Hospital, Central South University, Changsha, China.; 3 Department of Biochemistry and Molecular Biology, School of Life Sciences, Central South University, Changsha 410078, Hunan Province, China.

**Keywords:** epilepsy, hippocampal sclerosis, reactive astrocytes, glutamate dysregulation, m6A methylation

## Abstract

**Rationale:** Epilepsy affects over 70 million people globally, with temporal lobe epilepsy with hippocampal sclerosis (TLE-HS) often progressing to a drug-resistant state. Recent research has highlighted the role of reactive astrocytes and glutamate dysregulation in epilepsy pathophysiology. This study aims to investigate the involvement of astrocytic xCT, a glutamate-cystine antiporter, and its regulation by the m6A reader protein YTHDC2 in TLE-HS.

**Methods:** A pilocarpine-induced epilepsy model in mice was used to study the role of xCT in reactive astrocytes. The expression of xCT and its regulation by YTHDC2 were assessed through various molecular and cellular techniques. Quantitative real-time polymerase chain reaction (qRT-PCR) and western blotting were used to measure mRNA and protein levels of xCT and YTHDC2, respectively; immunofluorescence was utilized to visualize their localization and expression in astrocytes. *In vivo* glutamate measurements were conducted using microdialysis to monitor extracellular glutamate levels in the hippocampus. RNA immunoprecipitation-qPCR (RIP-qPCR) was performed to investigate the binding of YTHDC2 to SLC7A11 mRNA, while methylated RNA immunoprecipitation-qPCR (MeRIP-qPCR) was performed to quantify m6A modifications on SLC7A11 mRNA. A dual-luciferase reporter assay was conducted to assess the effect of m6A modifications on SLC7A11 mRNA translation, and polysome profiling was employed to evaluate the translational efficiency of SLC7A11 mRNA. Inhibition experiments involved shRNA-mediated knockdown of SLC7A11 (commonly known as xCT) and YTHDC2 expression in astrocytes. Video-electroencephalogram (EEG) recordings were used to monitor seizure activity in mice.

**Results:** The xCT transporter in reactive astrocytes significantly contributes to elevated extracellular glutamate levels, enhancing neuronal excitability and seizure activity. Increased xCT expression is influenced by the m6A reader protein YTHDC2, which regulates its expression through m6A methylation. Inhibition of xCT or YTHDC2 in astrocytes reduces glutamate levels and effectively controls seizures in a mouse model. Specifically, mice with SLC7A11- or YTHDC2-knockdown astrocytes showed decreased glutamate concentration in the hippocampus and reduced frequency and duration of epileptic seizures.

**Conclusions:** This study highlights the therapeutic potential of targeting YTHDC2 and xCT in reactive astrocytes to mitigate epilepsy. The findings provide a novel perspective on the mechanisms of glutamate dysregulation and their implications in seizure pathophysiology, suggesting that modulation of YTHDC2 and xCT could be a promising strategy for treating TLE.

## Introduction

Epilepsy is a common neurological condition affecting over 70 million people worldwide 1,2. Approximately one-third of these patients develop drug-resistant epilepsy 3, despite treatment with multiple anti-epileptic drugs (AEDs) 4. This translates to increased risks of premature death, injuries, psychosocial disorder, and a reduced quality of life 3. Temporal lobe epilepsy (TLE) is a common type of drug-resistant epilepsy, often associated with hippocampal sclerosis (TLE-HS). The main pathological changes include severe neuronal loss, dispersion of granule cells, mossy fiber sprouting, and reactive gliosis 5-7. While invasive resective surgery of the epileptogenic zone can offer seizure control, approximately 40% of patients experience early or late surgical failures 8. Therefore, effective disease-modifying treatments are urgently needed to achieve seizure control in patients with TLE-HS.

Recent research highlights the critical role of astrocytes in epilepsy pathophysiology 9-12. In healthy brains, astrocytes maintain normal neuronal activity by modulating neurotransmitter trafficking, ion homeostasis, energy metabolism, and neuroprotection, ensuring the optimal nervous system functioning 13,14. However, in epileptic conditions, astrocytes transition to a reactive state characterized by significant dysfunction 15,16. Studies using animal models of epilepsy suggest that specific changes in reactive astrocytes contribute to the development and recurrence of seizures 17,18. This suggests that reactive astrocytes are crucial in epilepsy initiation and progression.

Emerging evidence implicates glutamate dysregulation as a core feature of TLE-HS pathology 19,20. Numerous reports have confirmed elevated glutamate levels in epilepsy 21-23. As the predominant excitatory neurotransmitter, increased synaptic glutamate directly enhances neuronal excitability and seizure induction 24. Moreover, elevated extracellular glutamate likely plays a more pivotal role in seizures recurrence 23. High extracellular glutamate levels maintain neurons in a state of abnormal excitability 25, increasing susceptibility to epilepsy. They may also reduce the threshold for abnormal neuronal activity, shortening the interval between seizures and intensifying their frequency 26. Astrocytes are essential for glutamate metabolism 27 and regulating glutamate levels through the "glutamate/gamma-aminobutyric acid (GABA) shuttle" 28. In this process, astrocytes take up excess glutamate from the synaptic cleft and convert it into glutamine, which is released back into the extracellular space. Neurons subsequently take up glutamine and convert it back into glutamate for reuse as a neurotransmitter 29. Additionally, astrocytes can release glutamate, influencing neuronal excitability and synaptic transmission 30,31. Despite these known functions, the precise mechanisms by which reactive astrocytes modulate glutamate levels and influence epilepsy pathogenesis remain unclear.

In this study, we investigate the role of the glutamate-cystine antiporter (system Xc-) in reactive astrocytes of epileptic brains and its contribution to elevated extracellular glutamate concentrations. We demonstrated that increased xCT expression in reactive astrocytes is driven by YTHDC2-mediated enhancement of translation efficiency. Through a series of *in vivo* and *in vitro* experiments, we provide evidence that targeting xCT and YTHDC2 in reactive astrocytes significantly reduces extracellular glutamate and mitigates seizure activity, offering novel therapeutic targets for epilepsy treatment.

## Results

### Elevated glutamate in patients with TLE and mouse models

**Figure 1:**
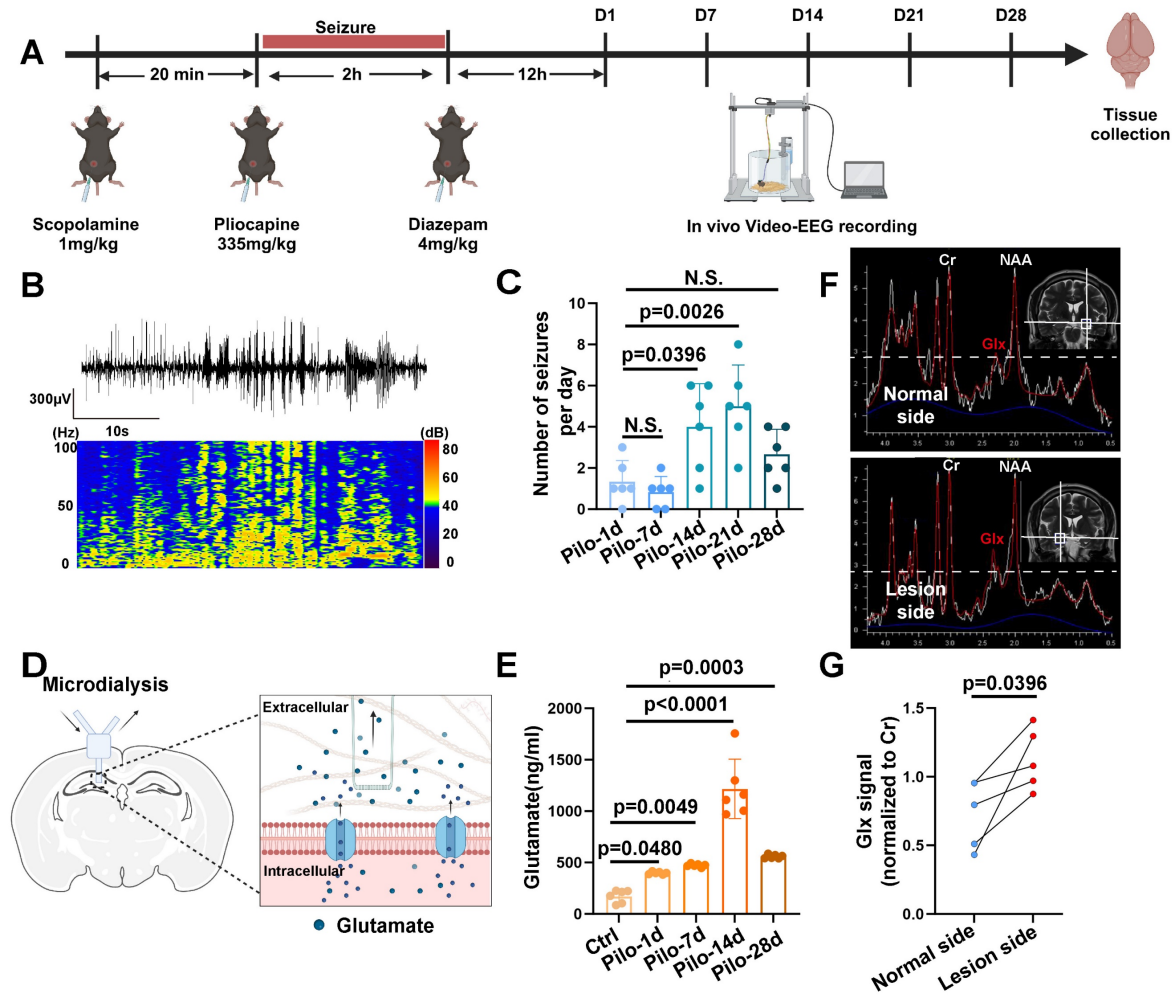
Glutamate dysregulation in patients with TLE and pilocarpine-induced mouse model. (**A**) Schematic illustration depicting the key steps involved in the pilocarpine-induced mouse model of epilepsy. (**B**) Representative images from video-electroencephalogram (EEG) recordings showing abnormal electrical discharges in the hippocampus of a pilocarpine-treated mouse brain. (**C**) Video-EEG recordings demonstrating a significant increase in the frequency of epileptic seizures starting 14 days after successful model induction with pilocarpine (*p* = 0.0026, *n* = 6). (**D**) Schematic illustration of the microdialysis technique used for extracting extracellular fluid from the mouse hippocampus. (**E**) The concentration of glutamate in the extracellular fluid of the mouse hippocampus increased on the first day after successful model establishment (*p* = 0.0480, *n* = 6), with a further, more pronounced increase observed on day 14 (*p* < 0.0001, *n* = 6). N.S. means no significant difference. (**F**) Magnetic resonance spectroscopy (MRS) of a representative patient with temporal lobe epilepsy (TLE) demonstrating Glx peaks in both hippocampi. The blue dashed line indicates the level of glutamate on the normal side. (**G**) In four patients with TLE, the intensity of the Glx signal was significantly higher on the lesion side compared with the normal side (*p* = 0.0396, *n* = 4). For B, C, D and E, data represent the mean ± standard error of the mean (SEM); one-way ANOVA with Bonferroni's post hoc test (B-E), two-sided paired Student's *t*-tests (F, G).

MRS data acquired at a 3.0 Tesla from patients with TLE-HS revealed a significant increase in the Glx signal within the lesioned side (*n* = 4) compared to the contralateral normal side, indicating abnormal glutamate levels (Figure 1F, G). Additionally, we employed a pilocarpine-induced TLE mouse model for further verification (Figure 1A, B). Video-EEG recordings confirmed successful model induction (modified Racine scale 4-5) followed by a latency period before the onset of spontaneous recurrent seizures (SRS) approximately 14 days post-induction (Figure 1C), consistent with previous reports 6. Glutamate levels in the extracellular fluid of the hippocampi, measured via microdialysis in mice, demonstrated a significant increase starting 14 days after model induction (Figure 1D, E). These findings not only corroborate our observations in patients with TLE but also suggest elevated glutamate concentrations within the hippocampus of mice with epilepsy during SRS.

### Excitatory amino acid transporters (EAATs) do not primarily cause elevated extracellular glutamate

**Figure 2:**
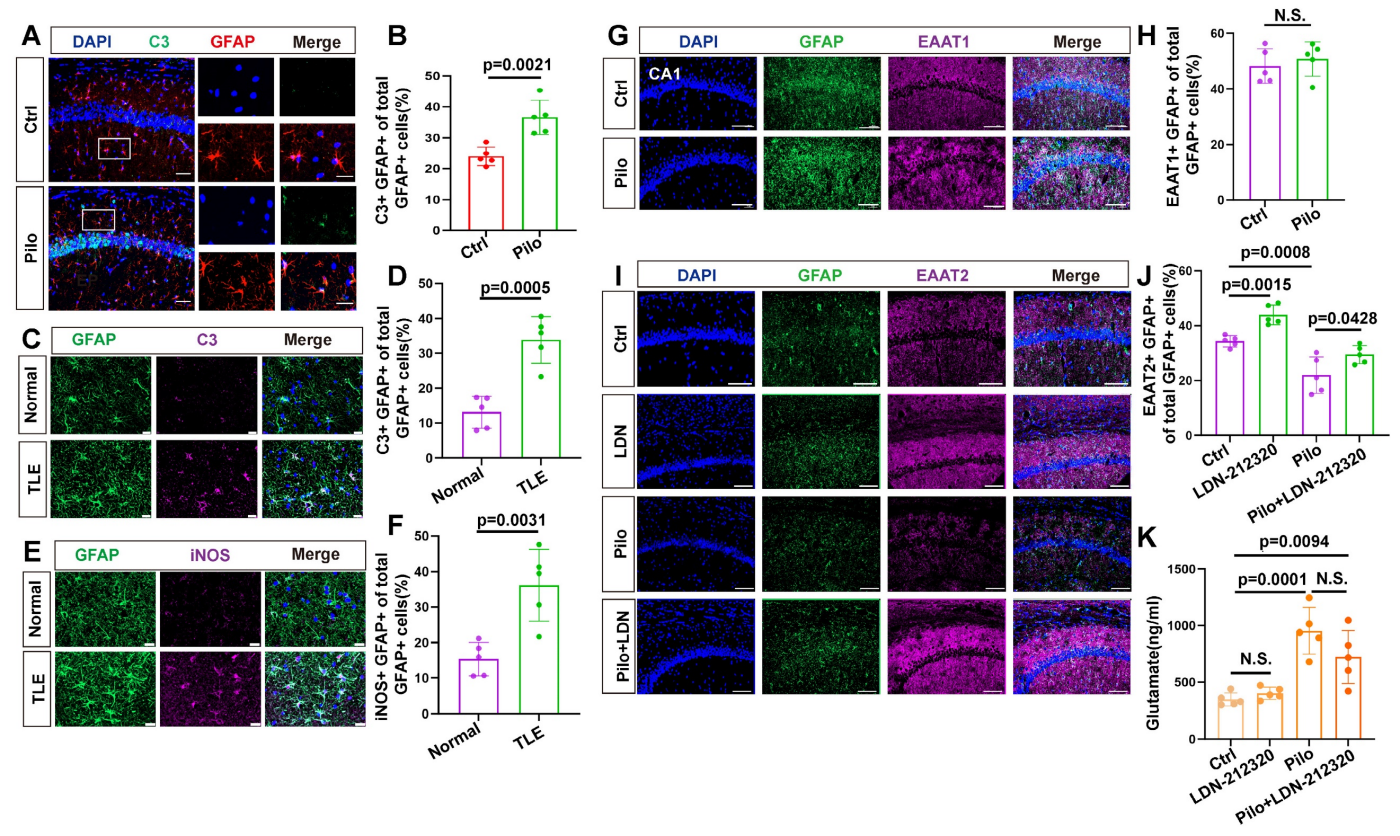
Immunofluorescence analysis of reactive astrocytes and EAAT expression in TLE. (**A, B**) Immunofluorescence staining reveals co-staining of astrocytes (identified by GFAP expression) and complement component 3 (**c3**) in the hippocampal CA1 area of both control and pilocarpine-treated mice (scale bar = 50 μm and 20 μm); Statistical analysis showed a significant increase in the proportion of C3-positive and GFAP-positive cells (activated astrocytes) among the total GFAP-positive cells in the pilocarpine-treated group (*p* = 0.0021, *n* = 5). (**C, D**) Immunofluorescence staining illustrated the expression of C3-positive astrocytes in normal brain tissue and the hippocampus of patients with TLE. A significantly increased proportion of C3-expressing astrocytes was observed in patients with TLE compared to the control group (*p* = 0.0005, *n* = 5). Scale bar = 50 μm. (**E, F**) Immunofluorescence staining displayed the expression of inducible nitric oxide synthase (iNOS) in astrocytes within the hippocampus of patients with TLE and normal brain tissue. The proportion of iNOS-expressing astrocytes was significantly higher in patients with TLE compared with the control group (*p* = 0.0031, *n* = 5). Scale bar = 50 μm. (**G, H**) Immunofluorescence staining of astrocytes within the CA1 area illustrates the expression of excitatory amino acid transporter 1 (EAAT1), a glutamate transporter (scale bar = 100 μm); No significant change was observed in the ratio of astrocytes expressing EAAT1 in the CA1 area of mice between the pilocarpine group and the control group (*n* = 5). (**I-K**) Immunofluorescence staining illustrates the expression of EAAT2 in astrocytes within the CA1 area across different treatment groups (scale bar = 100 μm); Compared with the control group, the proportion of EAAT2-positive astrocytes increased in the LDN-21230 treated group (*p* = 0.0115, *n* = 5), decreased in the pilocarpine group (*p* = 0.0008, *n* = 5), and increased in the group treated with LDN-21230 after pilocarpine compared with the pilocarpine-alone group (*p* = 0.0428, *n* = 5); glutamate concentrations in the extracellular fluid extracted by microdialysis from various treatment groups showed a significance increase in the pilocarpine-treated group compared with the control group (*p* = 0.0001, *n* = 5), with no significant difference observed after subsequent treatment with LDN-21230. two-sided unpaired Student's *t*-tests (B, D, F, H), two-way ANOVA with Bonferroni's post hoc test s (J, K).

Next, we examined the reactive state of astrocytes in patients with TLE and the mouse model. Immunohistochemical analysis of hippocampal tissue from patients with TLE, revealed an increase in complement component 3 (C3) and inducible nitric oxide synthase (iNOS) (markers of reactive astrocytes) within astrocytes (Figure 2C-F), suggesting potential astrocyte dysfunction during epileptic episodes. Similarly, in the hippocampal region of epileptic mice, significantly increased C3 levels were observed in the astrocytes compared with controls (Figure 2A, B).

Excitatory amino acid transporters (EAATs) play a critical role in seizure controls by removing glutamate from the synaptic cleft 21,32. However, their contribution to elevated extracellular glutamate levels remained unclear. Therefore, we investigated the expression of EAAT1 and EAAT2 in the mouse model (Figure 2G, I). EAAT1 expression in epileptic mouse hippocampal astrocytes showed no significant difference from controls (Figure 2H). However, EAAT2 expression was reduced (Figure 2J). To determine if the decrease in EAAT2 contributed to elevated extracellular glutamate levels in the epileptic mouse hippocampus, we used the EAAT2 activator LDN-212320 to restore EAAT2 immunoreactivity in hippocampal astrocytes (Figure 2I, J). Notably, extracellular glutamate levels in the hippocampus did not significantly decrease (Figure 2K), indicating that the rise in extracellular glutamate concentration in the epileptic mouse hippocampus was not solely due to reduced EAAT2 expression.

### System Xc- contributes to elevated glutamate *in vitro*

**Figure 3:**
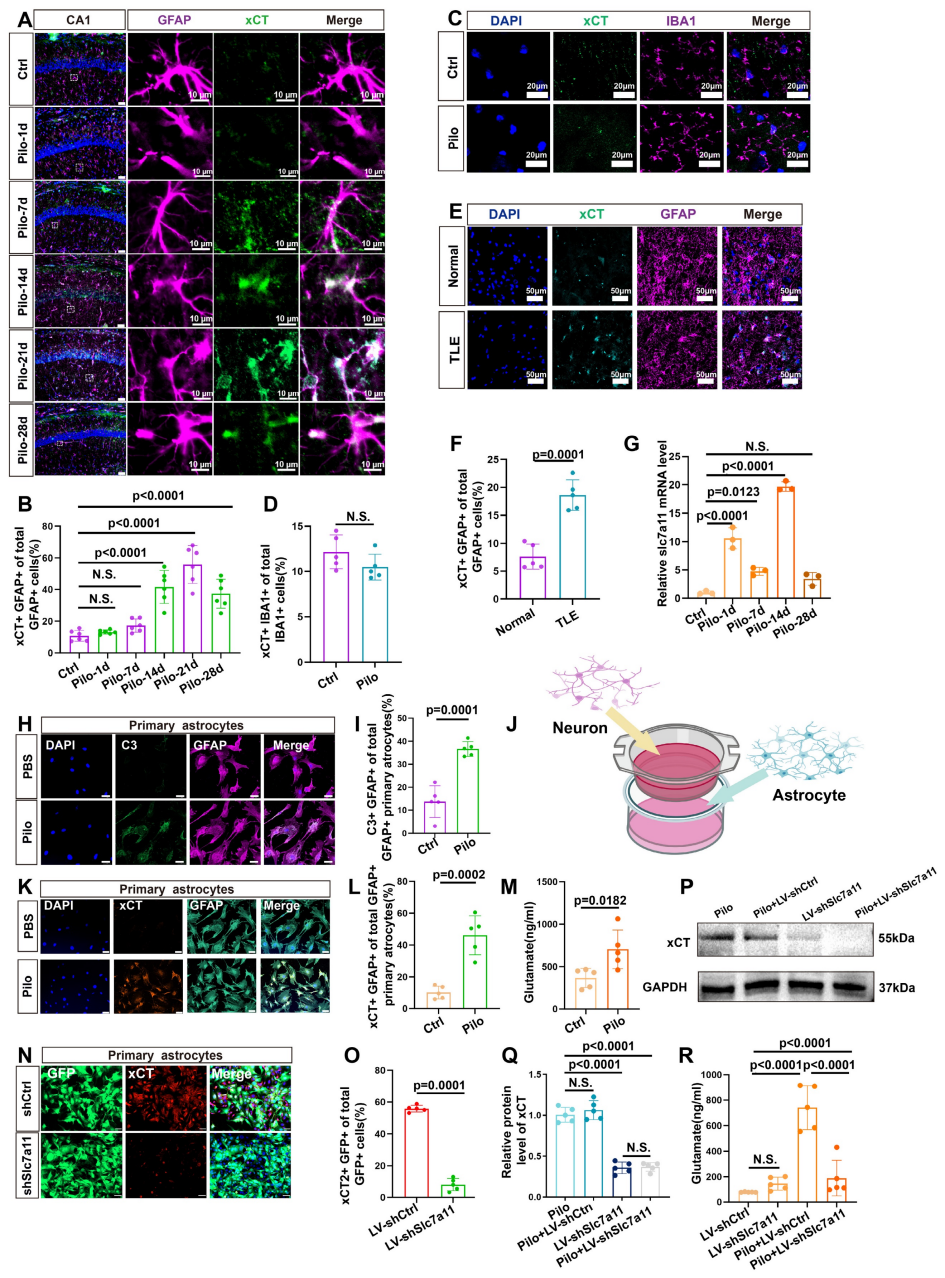
Expression of xCT in astrocytes and its impact on extracellular glutamate. (**A**) Immunofluorescence staining demonstrated the co-localization of astrocytes (identified by GFAP expression) and xCT in the CA1 area of mice at various time points following pilocarpine-induced epilepsy modeling (scale bar = 50 μm and 10 μm). (**B**) Statistical analysis revealed that compared with the control group, the proportion of astrocytes co-expressing xCT in the CA1 area did not significantly increase 1 or 7 days after epilepsy modeling. However, a marked increase was observed by day 14, 21 and 28 (*p* < 0.0001, *n* = 6). (**C**) Immunofluorescence staining displayed co-localization of microglia (IBA1-positive) and xCT in mice treated with pilocarpine compared with the control group (scale bar = 20 μm). (**D**) The proportion of microglia expressing xCT showed no significant difference between the pilocarpine-treated mice and the control group (*n* = 5). (**E**) Immunofluorescence staining displayed the co-localization of astrocytes and xCT in the hippocampus of patients with TLE and normal brain tissue (scale bar = 50 μm). (**F**) The proportion of astrocytes expressing xCT in the hippocampal tissue of patients with TLE significantly increased compared with the control group (*p* = 0.0001, *n* = 5). (**G**) Quantitative real-time polymerase chain reaction (qPCR) results indicated a significant increase in SLC7A11 mRNA expression (encoding xCT) 14 days after epilepsy modeling compared with the control group (*p* < 0.0001, *n* = 3). (**H**) Immunofluorescence staining of primary astrocytes demonstrates the expression of C3 in both control and pilocarpine-treated neurons co-culture groups (scale bar = 50 μm). (**I**) The proportion of primary astrocytes expressing C3 increased significantly in the pilocarpine-treated group compared with the control group (*p* = 0.0001, *n* = 5). (**J**) Schematic diagram of neuron-astrocyte co-culture. (**K**) Immunofluorescence of primary astrocytes showed the expression of xCT in both control and pilocarpine-treated groups (scale bar = 50 μm). (**L**) The proportion of primary astrocytes expressing xCT increased in the pilocarpine-treated group (*p* = 0.0002, *n* = 5). (**M**) Glutamate concentrations in the extracellular fluid of primary astrocytes from various treatment groups revealed an increase in the pilocarpine-treated group compared with the control group (*p* = 0.0182, *n* = 5). (**N, O**) Immunofluorescence staining showed the effect of SLC7A11 knockdown using short hairpin RNA (shRNA) carried by a lentivirus expressing green fluorescent protein (GFP) (*p* = 0.0001, *n* = 5). (scale bar = 50 μm). (**P, Q**) Western blot analysis revealed the expression levels of xCT protein in different groups after knockdown. Following the SLC7A11 knockdown, the relative expression level of xCT protein decreased compared with the control group (*p* = 0.0001, *n* = 5), with no significant change observed after pilocarpine treatment. N.S. means no significance. (**R**) The concentration of glutamate in the extracellular fluid of primary astrocytes significantly decreased in the SLC7A11 knockdown group compared with the vector control group (*p* < 0.0001, *n* = 5). N.S. means no significance; two-sided unpaired Student's *t*-tests (D, F, I, L M, O), one-way ANOVA with Bonferroni's post hoc test (B, G, Q, R).

Further investigations focused on the expression of xCT (heavy chain subunits of system Xc-, a glutamate-cystine antiporter), in murine hippocampal astrocytes. Compared to controls, a marked increase in xCT expression was detected in the astrocytes of epileptic mouse hippocampus (Figure 3A, B, and S1). In addition, no significant difference was observed in the co-localization of IBA1 (microglia marker) and xCT between the control and epileptic group (Figure 3C, D). Further, xCT expression did not significantly increase during the first week after induction of the epilepsy model but exhibited a significant upsurge from 14 days after pilocarpine-induced epilepsy (Figure 3A, B). This increase in xCT (SLC7A11) levels was further validated through qPCR and western blotting analyses (Figure 3G, and 6I, J). Additionally, examination of xCT expression in the hippocampi of patients with TLE and normal controls, revealed a significant increase in xCT within astrocytes of patients with TLE (Figure 3E, F). These findings collectively highlight the upregulation of xCT expression in hippocampal astrocytes of patients with epilepsy and mouse models.

Next, we explored the impact of xCT on extracellular glutamate levels in an *in vitro* model. We employed neuron-astrocyte co-cultures to simulate an *in vitro* epilepsy model, with pilocarpine used to stimulate neurons and induce astrocyte activation (Figure 3J). In the pilocarpine group, an increased proportion of astrocytes expressed the activation marker C3 (Figure 3H, I), accompanied by a significant rise in glutamate concentrations compared with the control group (Figure 3M). Concurrently, xCT expression levels were assessed and found to be a substantially increased in the pilocarpine group (Figure 3K, L). Subsequently, primary mouse astrocytes were transfected with lentivirus carrying shRNA to knock down SLC7A11 expression. In the SLC7A11-shRNA group, xCT expression in primary astrocytes was markedly suppressed (Figure 3N-Q, and S3), resulting in a noticeable reduction in extracellular glutamate concentrations (Figure 3R). These *in vitro* results validate the potential role of xCT in elevating extracellular glutamate levels, suggesting that its knockdown can effectively decrease extracellular glutamate concentrations.

### xCT knockdown reduces glutamate levels and seizure activity *in vivo*

**Figure 4:**
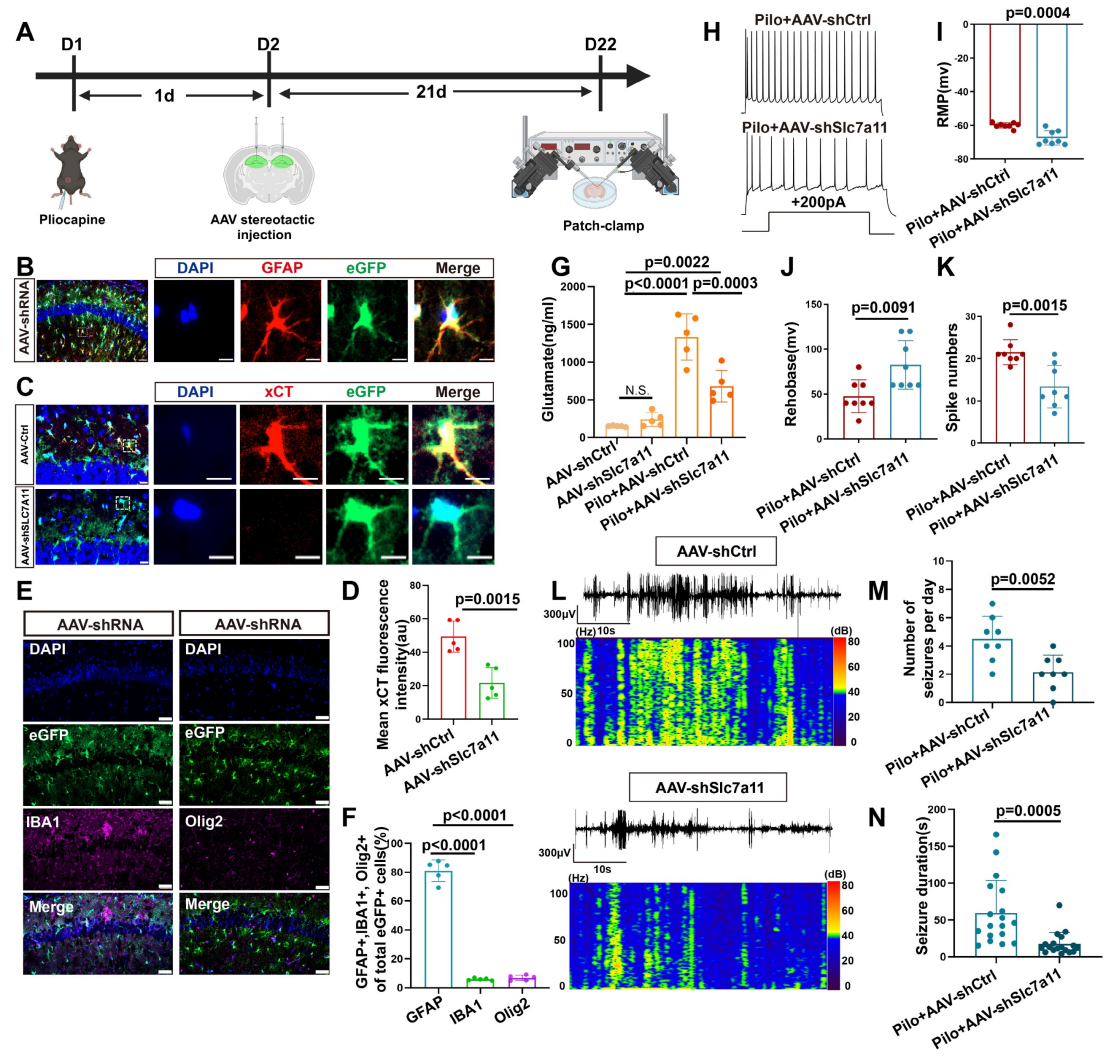
Targeted knockdown of SLC7A11 in astrocytes reduces xCT expression and seizure activity in a mouse model of epilepsy. (**A**) Schematic illustration of bilateral hippocampal injection in mice with adeno-associated virus 9 (AAV9) carrying eGFP to knockdown SLC7A11. (**B**) Immunofluorescence staining revealed the expression of eGFP in astrocytes within the CA1 area of the mouse hippocampus, scale bar = 50 μm and 5 μm; (**C, D**) Immunofluorescence staining demonstrates a significant decrease in xCT protein fluorescence intensity in the SLC7A11 knockdown group compared with the control group (*p* = 0.015, *n* = 5), indicating reduced xCT expression in astrocytes (scale bar = 20 μm and 5 μm). (**E, F**) Immunofluorescence staining for eGFP-positive cells co-stained with IBA1 (marker for microglia) and OLIG2 (marker for oligodendrocytes) revealed that most eGFP-positive cells co-localize with GFAP (marker for astrocytes), with only a minority co-staining with IBA1 (*p* < 0.0001, *n* = 5) or OLIG2 (*p* < 0.0001, *n* = 5), indicating predominant astrocyte targeting (scale bar = 50 μm). (**G**) Changes in glutamate concentration in the hippocampal extracellular fluid show a significant reduction in the SLC7A11 knockdown group compared with the vector control group (*p* = 0.0003, *n* = 5). (**H**) Whole-cell patch-clamp recordings of hippocampal neuronal activity in epileptic mice treated with AAV carrying control shRNA (AAV-ctrl-shRNA, *n* = 8) and AAV carrying SLC7A11 shRNA, *n* = 8). (**I-K**) Measurements of resting membrane potential (RMP) and rheobase indicate altered excitability levels in neurons. The SLC7A11 knockdown group shows a lower RMP compared with the vector control (*p* = 0.0004, *n* = 8), higher rheobase (*p* = 0.0091, *n* = 8), and fewer action potentials (spikes) fired (*p* = 0.0015, *n* = 8). (**L-N**) Representative EEG graphs for the SLC7A11 knockdown and vector control groups show that SLC7A11 knockdown reduces the average number of seizures per day (*p* = 0.0052, *n* = 8) and shortens seizure duration (*p* = 0.0005, *n* = 18), indicating a potential therapeutic effect against epilepsy. Two-sided unpaired Student's t-tests (D, I, J, K, M, N), one-way ANOVA with Bonferroni's post hoc test (F), and two-way ANOVA with Bonferroni's post hoc test (G).

To investigate the functional role of xCT *in vivo* regarding extracellular glutamate levels and epileptic seizures, we injected an adeno-associated virus (AAV) carrying the GFAP promoter and eGFP to bilaterally target SLC7A11 knockdown in the hippocampus of epileptic mice (Figure 4A). Immunofluorescence analysis revealed successful colocalization of eGFP with astrocytes, with minimal colocalization observed with ionized calcium-binding adaptor molecule (IBA-1; microglial marker) or oligodendrocyte transcription factor (OLIG2; oligodendrocyte marker) (Figure 4B, E, F), indicating efficient AAV-mediated transfection of astrocytes. Compared with the control group injected with AAV carrying control shRNA, a significant reduction in xCT protein expression fluorescence intensity was observed in the hippocampal astrocytes of mice injected with shRNA targeting SLC7A11 (Figure 4C, D). This demonstrates the effectiveness of shRNA in knocking down the SLC7A11 gene in astrocytes, leading to reduced xCT protein expression.

Mice in the SLC7A11-knockdown group showed a significant decrease in glutamate concentration compared with the control-AAV group (Figure 4G). Subsequent patch-clamp recordings of neurons adjacent to eGFP-expressing astrocytes revealed a significant decrease in resting membrane potential (RMP) and spike numbers, as accompanied by an increase in rheobase compared with control-AAV group (Figure 4H-K, and S7). These findings indicate a substantial reduction in neuronal excitability. Additionally, video-electroencephalogram (EEG) monitoring of the mice revealed that compared with the control-AAV group, knockdown of SLC7A11 significantly decreased the frequency and average duration of epileptic seizures in epileptic mice (Figure 4L-N), suggesting that xCT knockdown effectively controls epileptic seizures in this model.

### m6A methylation regulates of xCT expression in epilepsy

**Figure 5:**
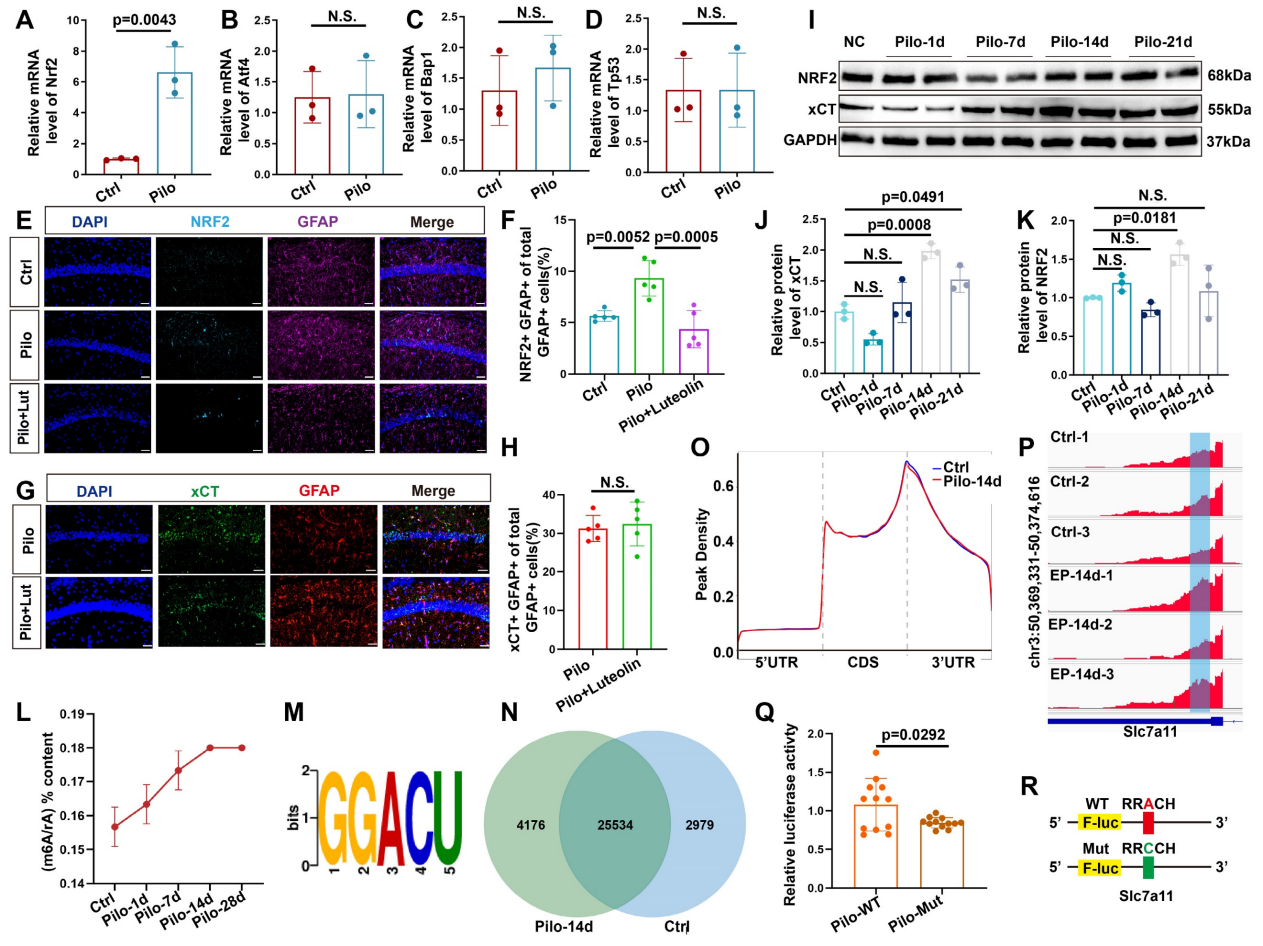
m6A methylation dynamics and its impact on SLC7A11 expression in epileptic mouse hippocampus. (**A-D**) Quantitative real-time polymerase chain reaction (qPCR) analysis revealed the relative expression of four transcription factors upstream of SLC7A11 in various groups of hippocampal tissues. NRF2 expression was significantly increased in the pilocarpine-treated group compared to the control group (*p* = 0.0043, *n* = 3). No statistically significant differences were observed in the expression of other transcription factors (*n* = 3). (**E, F**) Immunofluorescence analysis of astrocytes co-stained with NRF2 in the CA1 region of mice across different groups revealed an increase in the proportion of NRF2-positive astrocytes in the pilocarpine-treated mice compared with the control group (*p* = 0.0052, *n* = 5). Treatment with luteolin, an NRF2 inhibitor, resulted in a reduction of NRF2 expression (*p* = 0.0005, *n* = 5). Scale bar = 50 μm. (**G, H**) Immunofluorescence staining for xCT in astrocytes showed no significant difference between the pilocarpine and pilocarpine followed by luteolin treatment groups (*n* = 5). Scale bar = 50 μm. (**I-K**) Western blot analysis of the relative expression levels of NRF2 and xCT proteins at various time points showed an increase in NRF2 expression at 14 days (*p* = 0.0181, *n* = 3) and an increase in xCT expression after 14 days (*p* = 0.0008, *n* = 3). N.S. indicates no significance. (**L**) Liquid chromatography-mass spectrometry (LC/MS) was utilized to assess the degree of m6A methylation in mouse hippocampal tissue at various time points after pilocarpine injection (*n* = 3). (**M**) Using the Homer database, a common sequence motif was identified within the significantly differentially enriched m6A binding sites. (**N**) A Venn diagram illustrates the number of m6A methylated mRNAs in the control group compared with 14 days after epilepsy modeling. (**O**) In the control group and 14 days post-pilocarpine modeling, a metagene profile of significantly differentially enriched m6A methylation modification binding sites along normalized transcripts was generated. The profile is composed of three readjusted, non-overlapping segments: 5' untranslated region (5'UTR), coding sequence (CDS), and 3' untranslated region (3'UTR) (*n* = 3). (**P**) Integrative Genomics Viewer (IGV) tracks displayed MeRIP-seq read distribution along the CDS and 3'UTR of SLC7A11 mRNA. (**Q, R**) Schematic representation of wild-type or mutant m6A sites (adenine to cytosine mutation) in SLC7A11, fused with a dual-luciferase reporter gene. After transfecting primary astrocytes with wild-type or mutant SLC7A11 plasmids and stimulating with pilocarpine, the mutant group showed a decrease in luciferase intensity (*p* = 0.0292, *n* = 12). Two-sided unpaired Student's *t*-tests (A-D, H, Q), one-way ANOVA with Bonferroni's post hoc test (F), Kruskal-Wallis H test (J, K).

Since SLC7A11 mRNA is likely influenced by various transcription factors, we performed qPCR on four known regulators of SLC7A11 expression. Among these, only NRF2 was significantly increased (Figure 5A-D). Western blotting confirmed an increase in NRF2 protein levels 14 days post-model induction (Figure 5I, K). Previous reports suggest increased NRF2 expression in astrocytes of mice with epilepsy 33. Therefore, to determine if NRF2 upregulation was responsible for the increased xCT expression, we administered the NRF2 inhibitor luteolin to mice with epilepsy. Post-treatment, NRF2 expression decreased in astrocytes (Figure 5E, F); however, xCT levels remained largely unchanged (Figure 5G, H).

Given that the increase in xCT expression began 14 days after pilocarpine-induced modeling, we hypothesized that a post-transcriptional regulatory mechanism might be involved in this delayed increase. As m6A modification is the most common form of post-transcriptional regulation 34, we first examined m6A levels in the hippocampal tissue of mice with epilepsy. Following model induction, a gradual increase in the m6A/rA ratio was observed (Figure 5L-N, and S4A), indicating a potential rise in the m6A/rA ratio methylation modifications. Subsequent MeRIP-seq analysis in epileptic and control mice revealed that m6A modifications were predominantly located within the 3' untranslated region (3'UTR; Figure 5O). Therefore, we investigated whether SLC7A11 in hippocampal astrocytes of mice with epilepsy undergoes m^6^A modification. Notably, m^6^A modifications on SLC7A11 were significantly higher in mice with epilepsy compared with controls (Figure 5P and S4B). To further determine if m^6^A modifications contribute to the increased expression of SLC7A11, we engineered a luciferase reporter gene carrying potential m^6^A modification sites within the CDS and 3′UTR regions of SLC7A11 (Figure S2). Adenosine bases were mutated to cytosine in these sites (Figure 5R). Results showed that the mutated primary astrocytes exhibited significantly reduced luciferase activity compared with the wild type (Figure 5Q). This suggests that m^6^A methylation of SLC7A11 might be a key factor in elevating its expression levels.

### YTHDC2 mediates increased xCT expression

**Figure 6:**
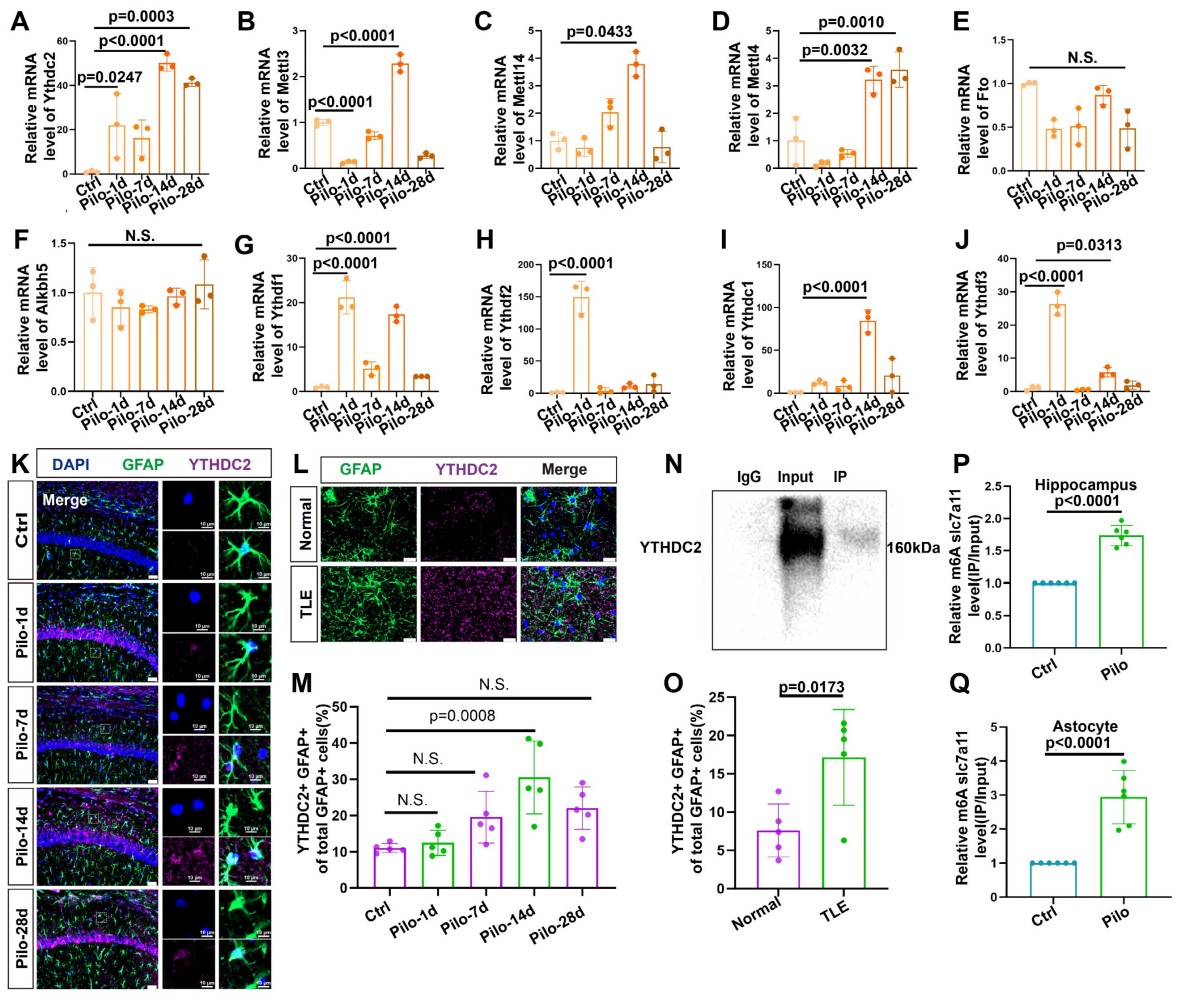
Upregulation of YTHDC2 in astrocytes following pilocarpine-induced epilepsy in mice. (**A-J**) Quantitative real-time polymerase chain reaction (qPCR) results revealed no significant changes in the relative mRNA expression levels of key m6A-related proteins at different time points (*n* = 3). N.S. means no significance. (**K, M**) Immunofluorescence staining revealed the expression of YTHDC2 in astrocytes within the mouse hippocampus at various time points following pilocarpine-induced epilepsy modeling. A significant increase in YTHDC2 expression was observed on day 14 compared with the control group (*p* = 0.0008, *n* = 5). Scale bar = 50 μm and 10 μm. (**L, O**) Immunofluorescence staining showed the expression of YTHDC2-positive astrocytes in normal brain tissue and the hippocampus of patients with TLE. The proportion of YTHDC2-expressing astrocytes was significantly higher in patients with TLE compared to the control group (*p* = 0.0173, *n* = 5). Scale bar = 50 μm. (**N, P, Q**) Representative western blot image from a YTHDC2-RNA immunoprecipitation (RIP) assay; Significant increases were observed in the binding of YTHDC2 to SLC7A11 mRNA in both the hippocampus of mice treated with pilocarpine (*p* < 0.0001, *n* = 6) and primary astrocytes co-culture with pilocarpine treated neurons (*p* < 0.0001, *n* = 6) compared with the control group. N.S. means no significance; one-way ANOVA with Bonferroni's post hoc test (A-J, M), two-sided unpaired Student's *t*-tests (O, P, Q).

We conducted qPCR analyses on hippocampal tissue from mice with epilepsy to identify potential proteins regulating the m6A methylation of SLC7A11 (Figure 6A-J). We found a consistent and dramatic increase in YTHDC2 at different time points after epilepsy induction (Figure 6A). Additionally, the number of astrocytes colocalized with YTHDC2 showed a marked increase in mice with epilepsy (Figure 6K, M). A significant rise in the proportion of YTHDC2-positive astrocytes was similarly observed in the hippocampal tissue of patients with TLE (Figure 6L, O). Further investigation through the YTHDC2-RIP assay confirmed the binding of YTHDC2 to SLC7A11 mRNA. This binding was significantly increased within the *in vivo* and *in vitro* models (Figure 6N, P, Q**)**. These findings suggest that YTHDC2 may act as the m6A reader protein, regulating SLC7A11 expression under epileptic conditions.

To exclude the potential influence of pilocarpine on YTHDC2 and m6A methylation, we conducted *in vitro* experiments using kainic acid (KA)—another common epilepsy modeling drug. YTHDC2 and METTL3 levels also increased in primary astrocytes after treatment with KA. Hence, the increase in YTHDC2 was consistent across different epilepsy models (Figure S6).

### YTHDC2 promotes xCT expression by enhancing translational efficiency

**Figure 7:**
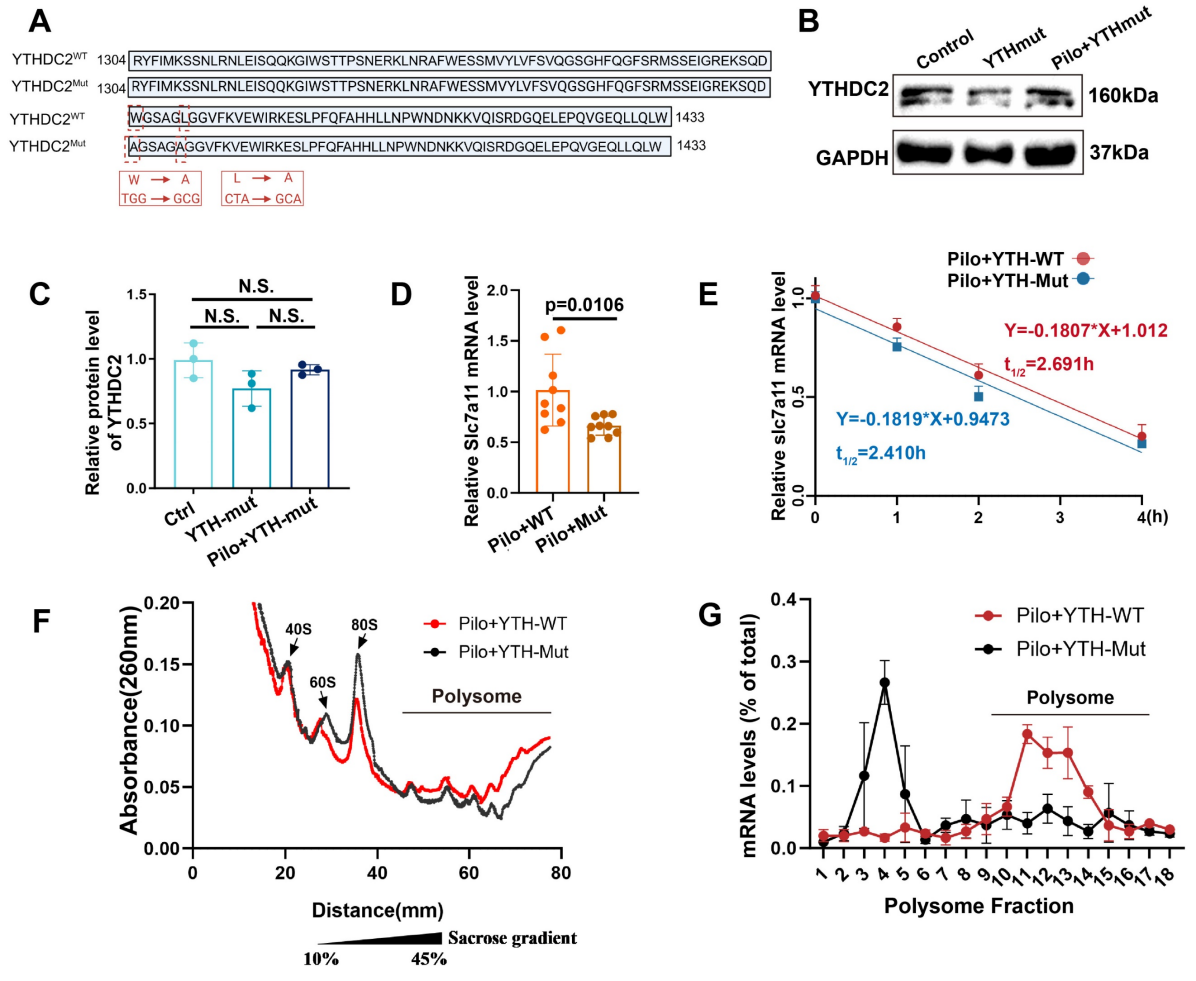
YTHDC2 mediates increased xCT expression by increasing SLC7A11 mRNA translation efficiency. (**A**) The YTH domain of the YTHDC2-Mut allele (indicated by the red dashed line, required for m6A interaction) exhibits changes in nucleotides and amino acids compared with the wild-type sequence. (**B, C**) Western blot analysis showed no significant differences in YTHDC2 expression among various groups of in vitro astrocytes (*n* = 3). (**D**) Quantitative real-time polymerase chain reaction (qPCR) revealed a significant decrease in the relative expression of SLC7A11 mRNA in primary astrocytes treated with pilocarpine in the YTH-Mut group compared to the YTH-WT group (*p* = 0.0106, *n* = 8). (**E**) YTH-WT and YTH-Mut astrocytes were treated with actinomycin D (ActD, 5 μg/mL) for specified durations. The expression of SLC7A11 mRNA was examined through qRT-PCR (*n* = 3). (**F, G**) Sucrose gradient-based polysome profiling was performed on YTH-WT and YTH-Mut astrocytes. The quantification of SLC7A11 mRNA in each ribosomal fraction was performed by qRT-PCR and plotted as a percentage of the total (*n* = 3). N.S. indicates no significance; Kruskal-Wallis H test (C), two-sided unpaired Student's *t-*tests (D).

The YTHDC2 protein contains several functional domains, with the YTH domain critical for its activity. To ascertain whether YTHDC2 regulates SLC7A11 expression in astrocytes of mice with epilepsy through its YTH domain in an m^6^A-dependent manner, we constructed a plasmid encoding a mutant YTH domain (YTH-mut) based on previous research 35. This mutation disrupts the function of the YTH domain without affecting other protein domains (Figure 7A). Following pilocarpine stimulation, a significant reduction in the relative abundance of SLC7A11 mRNA was observed in astrocytes transfected with the YTH-mut plasmid compared with wild-type YTHDC2 (YTH-WT; Figure 7B-D), indicating that YTHDC2 mediates the regulation of SLC7A11 expression through its YTH domain.

To further investigate the impact of YTHDC2 on SLC7A11 mRNA stability, we employed actinomycin D to inhibit RNA synthesis and monitored the decay of SLC7A11 mRNA over time (Figure 7E). No significant difference in mRNA stability was observed between the YTH-WT and YTH-mut groups, suggesting that YTHDC2 does not regulate SLC7A11 expression by increasing its mRNA stability. We then used cycloheximide to block translation, followed by polysome profiling, to examine the effect of YTHDC2 on SLC7A11 mRNA translational efficiency. Results revealed a significant decrease in SLC7A11 mRNA translation efficiency in the YTH-mut group compared with the YTH-WT group (Figure 7F, G). These findings suggest that YTHDC2 enhances the translational efficiency of SLC7A11 mRNA under epileptic conditions, ultimately leading to increased xCT protein expression.

### Therapeutic potential of targeting YTHDC2 in epilepsy

**Figure 8:**
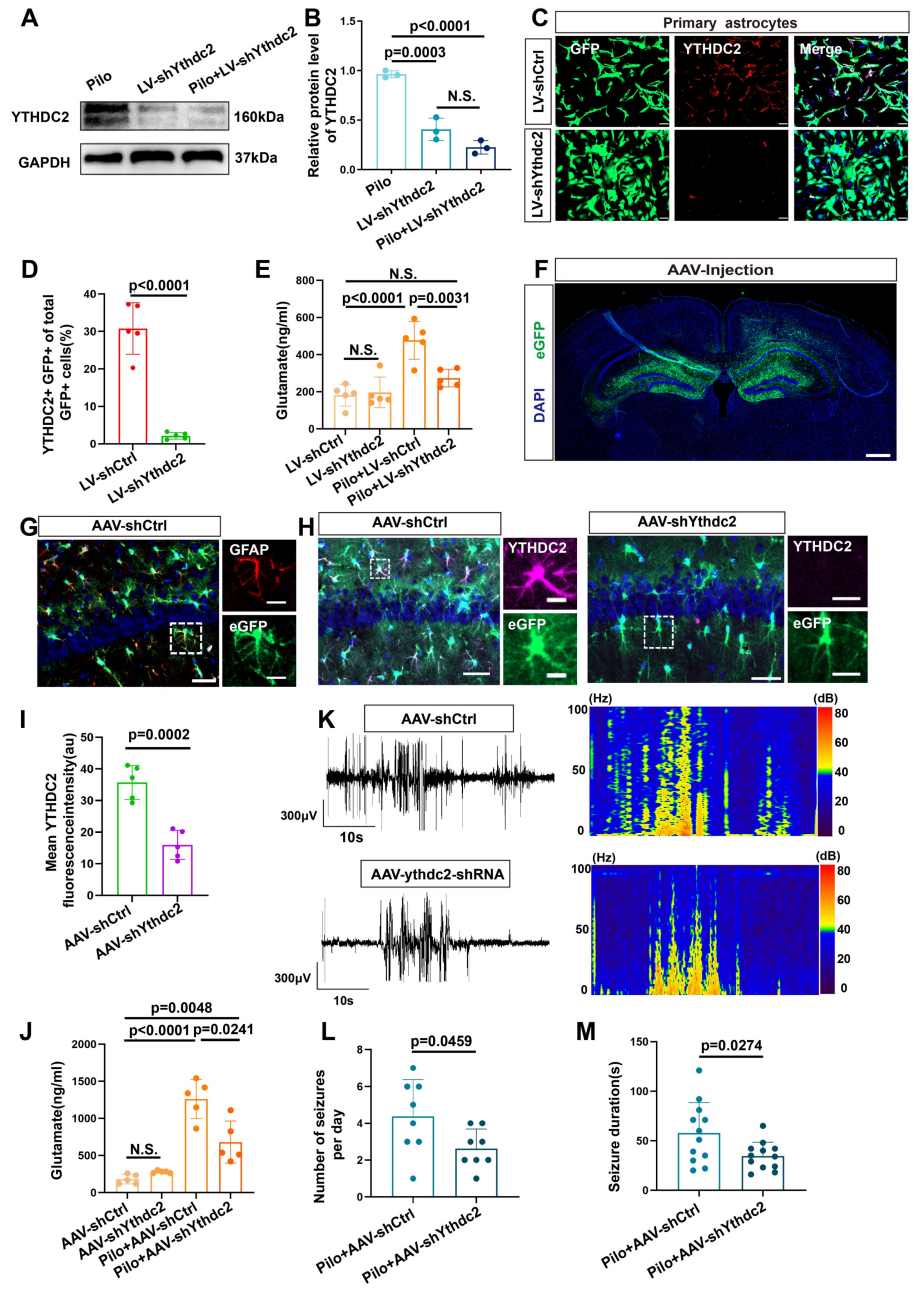
Reduction of YTHDC2 expression in astrocytes decreases extracellular glutamate levels and seizure activity in a mouse model of epilepsy. (**A, B**) Western blot analysis revealed the expression levels of YTHDC2 protein in various groups of *in vitro* astrocytes. The YTHDC2 knockdown group showed a reduced relative expression of YTHDC2 protein compared with the control group (*p* = 0.0003, *n* = 3). After stimulation with pilocarpine, the relative expression of YTHDC2 showed no significant difference compared to the knockdown group alone (*n* = 3). (**C, D**) Immunofluorescence staining demonstrated the knockdown of YTHDC2 using shRNA carried by a lentivirus expressing GFP (*p* < 0.0001, *n* = 5), scale bar = 50 μm. (**e**) *In vitro* measurement of glutamate concentration showed a significant decrease in the YTHDC2 knockdown group compared with the vector control group (*p* = 0.0031, *n* = 5). (**F**) Representative image of eGFP expression 21 days post bilateral hippocampal injection of AAV, scale bar = 500 μm. (**G**) Immunofluorescence staining revealed the expression of eGFP in astrocytes within the CA1 area of the mouse hippocampus (scale bar = 50 μm and 5 μm). (**H, I**) Immunofluorescence staining showed a significant decrease in YTHDC2 protein expression in astrocytes of the YTHDC2 knockdown group compared with the control group (*p* = 0.002, *n* = 5), scale bar = 50 μm and 5 μm. (**J**) The concentration of glutamate in the extracellular fluid of the hippocampus across different treatment groups showed a significant decrease in the YTHDC2 knockdown group compared with the vector group after pilocarpine treatment (*p* = 0.0241, *n* = 5). (**K-M**) Representative EEG graphs for the YTHDC2 knockdown and vector control groups showed a decrease in the average number of seizures per day (*p* = 0.0459, *n* = 8) and a shortening of seizure duration in the YTHDC2 knockdown group (*p* = 0.0274, *n* = 12). N.S. means no significance; Kruskal-Wallis H test (B), two-sided unpaired Student's *t-*tests (D, I, L, M), two-way ANOVA with Bonferroni's post hoc test (E, J).

We next investigated the effect of YTHDC2 knockdown in an *in vitro* model (Figure 8A-D). Knockdown of YTHDC2 decreased xCT expression (Figure S5C, D) and subsequently reduced the extracellular glutamate concentration (Figure 8E). To explore the effect of YTHDC2 knockdown on epileptic seizures, we injected an AAV carrying the GFAP promoter and eGFP into the bilateral hippocampus of mice with epilepsy to specifically reduce YTHDC2 expression in astrocytes (Figure 8F).

Colocalization of eGFP with astrocytes (identified by GFAP expression) was observed in the hippocampal region (Figure 8G). Furthermore, a significant decrease in YTHDC2 fluorescence intensity was observed in the eGFP-positive cells of the knockdown group compared with the control group (Figure 8H, I). Additionally, xCT fluorescence intensity was significantly reduced in the YTHDC2 knockdown group (Figure S5A, B). Subsequently, measurement of extracellular glutamate concentrations in the hippocampal region, revealed a significant reduction in the YTHDC2 knockdown group (Figure 8J). Finally, video-EEG monitoring demonstrated a decrease in both the number and duration of epileptic seizures in the YTHDC2 knockdown group (Figure 8K-M). These findings collectively indicate that YTHDC2 knockdown can effectively lower extracellular glutamate levels and control epileptic seizures.

## Discussion

Our study demonstrates that increased xCT expression in reactive astrocytes is a key contributor to elevated extracellular glutamate levels in TLE, ultimately leading to increased neuronal excitability and seizures. It is well-established that patients with epilepsy exhibit high glutamate concentrations in the epileptic foci 36. However, the underlying mechanisms remain elusive. We employed high-field MRS at 3.0 Tesla to analyze glutamate levels in patients with TLE-HS, using the contralateral normal side as a control. Our findings revealed significantly higher glutamate levels on the lesioned side compared with the normal side, consistent with previous MRS studies conducted on different brain regions in patients with epilepsy 37,38. This suggests that elevated glutamate levels within epileptogenic foci of patients. These observations were further validated in a pilocarpine-induced mouse model of epilepsy, where a notable increase in glutamate concentration was observed in the hippocampus, aligning with previous reports 23. Notably, approximately 14 days post-model induction, a significant increase in spontaneous epileptic seizures coincided with elevated glutamate levels, suggesting a potential role of increasing glutamate concentrations in promoting seizure activity.

The role of reactive astrocytes in epilepsy has garnered increasing attention, primarily due to their critical involvement in regulating glutamate metabolism 39,40. We first examined the activation status of astrocytes in patients with epilepsy and mouse models. In both cases, reactive astrocytes was observed, with significant increased abundance compared with controls, corroborating prior findings 11,41-43. Regarding the abnormal regulation of glutamate metabolism by reactive astrocytes, previous studies have reported changes in the expression levels of EAATs, particularly EAAT1 and EAAT2 44-46. These changes could lead to glutamate accumulation in the synaptic gap due to diminished clearance. While we found no significant statistical difference in EAAT1 levels, a notable decrease in EAAT2 expression was detected. However, activating EAAT2 expression did not significantly reduce extracellular glutamate levels. Although conditional EAAT2 knockout in the astrocytes of mice increases extracellular glutamate levels 47, suggesting that EAAT2 plays a role in controlling glutamate concentration, other studies have reported that overexpressing EAAT2 in epilepsy mice only reduces glutamate levels during the acute phase (within 3 days of modeling), not in the chronic phase (28 days post-modeling) 48. One possible explanation for this discrepancy lies in the specific role of astrocytic EAAT2. It is believed that EAAT2 primarily regulates synaptic glutamate release through rapid binding and high-density expression around synapses 49,50. Notably, our research indicates that the increase in extracellular glutamate concentration in mice with epilepsy does not peak in the acute phase immediately following model induction but rather reaches its highest level on day 14. Similarly, the frequency and duration of seizures begin to increase at this time and peak approximately 21 days after modeling. This suggests a potential association between elevated extracellular glutamate concentrations and recurrence of seizures during this period.

We next investigated the potential role of increased glutamate release by astrocytes. xCT, a crucial component of the system Xc-, has been implicated in epilepsy 51,52. xCT plays a crucial physiological role in maintaining the cellular redox balance by facilitating the exchange of extracellular cystine and intracellular glutamate 53. Additionally, this antiporter is essential for glutathione synthesis—a major antioxidant that protects cells from oxidative stress 54. Hence, by regulating extracellular glutamate levels, xCT also modulates neurotransmitter activity, preventing excitotoxicity. Previous studies have shown that knocking out xCT in the Theiler's murine encephalomyelitis model of viral-induced epilepsy reduces neuronal damage and leads to astrocytes hypertrophy 51. Notably, this does not decrease the frequency of seizures in this model. Another study found an increase in xCT expression levels in mice induced by pilocarpine 52. Additionally, in a model of self-sustained status epilepticus induced by electrical kindling in mice, knocking out xCT reduced the frequency of seizures 52. However, there was limited research on the variations in xCT expression levels on the increased extracellular glutamate concentrations.

Thus, we evaluated xCT transporter expression, revealing significant increases in patients with TLE and mice with epilepsy. Furthermore, in the pilocarpine-induced mouse model, xCT expression did not significantly increase in the early stages post-induction but showed a marked increase 14 days post-modeling, coinciding with a significant rise in spontaneous epileptic seizures 55. Similarly, an increase in xCT expression levels was observed in pilocarpine-induced primary astrocyte cultures. We also found that knocking down SLC7A11 (the gene encoding xCT) reduces extracellular glutamate concentration *in vivo* and *in vitro*. It also decreases the excitability of neighboring neurons and reduces seizures, suggesting potentially important roles for xCT in epilepsy.

xCT expression can be regulated by several transcription factors 56. To determine the possible reasons for the delayed increase in xCT expression 14 days post-modeling, we first investigated the involvement of several common transcription factors. However, their involvement was excluded. We then considered the possibility that the increased expression of xCT could be due to post-transcriptional regulation. For the first time, we report changes in the overall level of m^6^A methylation in the hippocampi of epileptic mice, with m^6^A levels progressively increasing following model induction. This suggests that m^6^A methylation might mediate pathophysiological changes in this epilepsy model. Indeed, m^6^A, the most common form of post-transcriptional regulation 34,57, plays a significant role in various physiological and pathological processes 58,59. Subsequent MeRIP-seq analysis of mouse hippocampal tissue revealed that m^6^A methylation was primarily located within the CDS and 3′UTR regions of transcripts, indicating its potential role in regulating translation and mRNA stability. Further examination of changes in m^6^A methylation levels, specifically in SLC7A11 mRNA, revealed upregulated m^6^A methylation modifications in both the CDS region and 3′UTR in the epilepsy group.

To determine whether m^6^A modifications in SLC7A11 were responsible for the increased expression observed, we constructed a luciferase reporter plasmid. We mutated potential m^6^A modification sites (adenine bases) in the SLC7A11 mRNA's CDS and 3′UTR regions to cytosine. Our findings suggest that m^6^A methylation modifications might be the cause of increased SLC7A11 expression, aligning with observations in various disease models 60,61.

YTHDC2 is an m6A reader protein that recognizes and binds to m^6^A-modified RNA, playing a critical role in RNA metabolism and influencing RNA stability and translation efficiency 34. We further identified YTHDC2 as the m^6^A reader protein specifically regulating the increased expression of SLC7A11. This finding is noteworthy given the observed increase in YTHDC2 expression in astrocytes of mice with epilepsy. Given that YTHDC2 possesses intrinsic helicase activity 35,62-64, we specifically introduced mutations into the YTH domain to impair its binding affinity for m6A, circumventing the influence of other structural domains. Contrary to observations in lung cancer 65, where YTHDC2 was reported to diminish SLC7A11 expression through reduced SLC7A11 mRNA stability, our findings reveal that YTHDC2 facilitates the upregulation of SLC7A11 expression by enhancing its translational efficiency in the epileptic condition. *In vitro* studies investigating SLC7A11 mRNA stability and translational efficiency through mutations in the YTH domain supported these observations. The quantity of SLC7A11 mRNA bound to polysomes significantly decreased after YTH domain mutation, indicating a significant drop in translational efficiency. By knocking down YTHDC2 *in vivo* and *in vitro*, we observed a decrease in xCT protein levels in astrocytes, accompanied by a reduction in glutamate levels. In the epilepsy mouse model, YTHDC2 knockdown also effectively controlled seizures, suggesting YTHDC2 as a potential target for epilepsy therapy. A recent study found differentially abundant m6A-associated proteins the peripheral blood of epileptic patients. Specifically, levels of YTHDC1 increased in patients with epilepsy, while those of YTHDC2 did not differ significantly 66. Additionally, another study using a PTZ mouse model reported significantly reduced m6A methylation levels in the dentate gyrus. Meanwhile, administering agonists to restore m6A methylation levels or upregulating METTL14/YTHDC1 expression effectively controlled epilepsy 67. These findings illustrate the complexity and diversity of m6A trends in epilepsy development. However, more in-depth investigations are warranted to explore and validate the effects of specific m6A-related proteins and their downstream targets in epilepsy.

In conclusion, our study unveils a novel mechanism by which increased xCT expression in reactive astrocytes contributes to elevated extracellular glutamate levels in TLE. This increase in xCT expression is primarily mediated by the m6A reader protein YTHDC2, which enhances translational efficiency through a post-transcriptional regulatory mechanism. Knockdown of xCT and YTHDC2 in reactive astrocytes effectively reduced glutamate concentrations and diminished seizure activity. These findings suggest that targeting xCT and YTHDC2 in reactive astrocytes could offer a novel therapeutic strategy for drug-resistant epilepsy. Future investigations could focus on further elucidating the precise mechanisms by which YTHDC2 regulates SLC7A11 translation. Additionally, exploring the therapeutic potential of targeting the m6A-YTHDC2-xCT pathway for the treatment of TLE holds significant promise.

## Methods

### Animals

Male C57BL/6J mice aged 6-8 weeks were purchased from SLAC Animal (Changsha, Hunan Province, China) and were in an environment with a temperature and humidity suitable for the animals, with a 12-h light/dark cycle. Neonatal 1-2-day C57 BL/6J mice were purchased from SLAC animal (Changsha, China) for primary astrocyte and primary neuron extraction. Animal experiments were conducted in accordance with the Guide for the Care and Use of Laboratory Animals of the National Institutes of Health and approved by the Animal Care and Use Committee of Xiangya Hospital, Central South University, Hunan Province (Approval No. 202103085). All animal experimental procedures were in accordance with the Guide for the Care and Use of Laboratory Animals of the National Institutes of Health.

### Magnetic resonance spectroscopy (MRS) for patients with TLE

MRS evaluations were performed on four patients with TLE-HS at Xiangya Hospital, after obtaining informed consent. The studies used a GE 3.0T (General Electric Company, Boston, MA, USA) scanner with an eight-channel head coil for data collection. Two-dimensional multivoxel proton MRS utilized parameters, such as TR/TE of 2000/32 ms, spectral width of 2000 Hz, and an average of four signals, incorporating water suppression and excitation. The scan covered a 100 mm × 100 mm field of view, focusing on a 64 mm × 32 mm volume of interest. Alignment was parallel to the axial T2-weighted images, perpendicular to coronal fluid-attenuated inversion recovery images, and included 1 mm high-resolution sagittal 3D T1-weighted imaging for voxel positioning.

### Human hippocampal tissue

Hippocampal tissues from individuals with epilepsy were collected with informed consent at the Department of Functional Neurosurgery, Xiangya Hospital, affiliated with Central South University. The tissues were taken from patients identified with TLE-HS. The ethical clearance for utilizing these tissues was approved by the Ethics Committee of Xiangya Hospital, Central South University, as recorded under the number 202401015. For comparative analysis, control tissues were obtained from the Department of Human Tissue and Anatomy at Xiangya Medical College of Central South University. These control specimens came from individuals who had donated their bodies for research and were confirmed to have no central nervous system disorders 68.

### Establishment of a TLE mouse model

Male C57 mice, aged 6 -8 weeks, were chosen to develop a model of TLE. Before inducing epilepsy, scopolamine hydrates at a dose of 1 mg/kg (MCE, USA) was administered intraperitoneally to each mouse to reduce peripheral side effects 55. This pre-treatment was succeeded by the intraperitoneal injection of pilocarpine at a dose of 335 mg/kg (Sigma, USA) 20 min later. The intensity of seizures was evaluated using the modified Racine scale 69. Seizures were terminated 2 h after their induction with a 4 mg/kg intraperitoneal injection of diazepam (Sigma). Following the establishment of the model, mice were given daily intraperitoneal injections of PBS, LDN-21230 (10 mg/kg, MCE) or luteolin (25 mg/kg, MCE) for ongoing investigations.

### Video-EEG recording

Mice were anesthetized using intraperitoneal injection of sodium pentobarbital (100 mg/kg) and subsequently positioned within a stereotaxic apparatus. Following cranial exposure, surface electrodes were implanted atop the murine frontal-parietal cortex. Electromyographic (EMG) electrodes were embedded beneath the dermis of the neck for the acquisition of EMG signals. Electrodes were then secured using dental cement. Post-operatively, the mice were housed for a week prior to model establishment and AAV stereotactic injection. Electrodes were connected to a data regulation and acquisition system (MX2, Data Sciences International, Inc) for the continuous recording of EEG activity over 24 h at various time points. EEG signals were analyzed using Ponemah v6.51 software (Data Sciences International, St. Paul, MN, USA) to identify electrographic epileptic events, defined by a minimum duration of 10 s at amplitudes twice that of baseline.

### Whole-cell patch-clamp

Whole-cell patch-clamp recordings were performed on hippocampal neurons with borosilicate glass pipettes (3-9 MΩ). Pipettes were filled with 120 mM potassium methylsulfate (20 mM) KCl, 2 mM MgCl_2_, 10 mM HEPES, 0.2 mM EGTA, 4 mM Na_2_-ATP, 0.3 mM GTP-Tris, and pH 7.25. During recording sessions, hippocampal neurons were visualized with an Olympus BX51WI microscope (Olympus Corporation, Tokyo, Japan). The bright fluorescence signal of eGFP-positive astrocytes was detected using a xenon light source and an MTI-Dage CCD camera (IR-1000, Meyer Instruments). Patch-clamp recordings were acquired with an Axon Multiclamp (700B, USA) and the signals were fed into a computer through a Digidata (1440A, USA) for data capture and analysis. Recordings of membrane potentials were low-pass filtered at 5 kHz and digitized at 10 kHz.

### *In vivo* glutamate measurement

To monitor glutamate levels *in vivo*, microdialysis was employed to sample the extracellular fluid of the mouse hippocampus. Under isoflurane anesthesia, a microdialysis probe (AI-4-02, Eicom) was stereotactically inserted into the hippocampus CA1 (anteroposterior = -2.0 mm; mediolateral = ± 1.8 mm, dorsoventral = -1.5 mm) according to a minimally invasive protocol 41. The probe was perfused with artificial cerebrospinal fluid at a flow rate of 1 μL/min to ensure the stable recovery of glutamate throughout the sampling period. Samples were collected every 20 min for a duration of 4 h to establish the baseline glutamate concentration. Collected samples were immediately frozen at -80 °C to prevent glutamate degradation. Prior to analysis, samples were thawed on ice and centrifuged to remove any particulate matter. The supernatant was then derivatized to enhance the sensitivity of glutamate detection by liquid chromatography-mass spectrometry (LC-MS). The LC-MS analysis utilized a reverse-phase chromatographic column along with a gradient elution of aqueous and organic solvents. Glutamate concentrations were quantified using a calibration curve established with known standards. The LC-MS method offers high sensitivity and specificity for measuring extracellular glutamate levels in hippocampal samples, facilitating the study of dynamic changes in neurotransmitter concentrations *in vivo*.

### *In vitro* glutamate measurement

Following the completion of treatments on primary astrocytes in each group, the supernatant was discarded, and the cells were washed with phosphate -buffered saline (PBS) to eliminate any remaining culture medium. Subsequently, the cells were incubated for 2 h in artificial cerebrospinal fluid containing glutamate. After the incubation, the glutamate concentration in the artificial cerebrospinal fluid was measure using LC-MS.

### AAV-mediated selective knockdown in astrocytes

AAV-mediated selective knockdown of astrocytic genes was achieved using AAV9-GFAP-SLC7A11/YTHDC2-shRNA-eGFP constructs acquired from GenScript. These constructs integrate the shRNA cassette into the pHB-AAV-GFAP-EGFP vector, which contains an eEGFP reporter gene. A scrambled shRNA sequence served as the control. AAV vectors were produced in HEK293T cells (CRL-3216, ATCC) using a tri-plasmid system comprising pAAV-RC, pHelper, and pHB-AAV-GFAP-EGFP vectors carrying either SLC7A11/YTHDC2 shRNA or control shRNA. The viral particle titers were determined by qPCR. Virus aliquots were stored at -80°C and thawed on ice before use.

Mice were anesthetized through intraperitoneal injection of pentobarbital sodium (100 mg/kg body weight) and secured in a stereotaxic. Constructs, such as AAV9-GFAP-SLC7A11-shRNA-eGFP, AAV9-GFAP-YTHDC2-shRNA-eGFP, and AAV9-GFAP-shRNA (NC)-eGFP were injected into the dorsal hippocampal region (2.0 mm posterior to the bregma, 1.5 mm lateral, and 1.5 mm ventral; 2 µL per hemisphere). To prevent backflow, the micropipette was left in place for 15 min after virus microinjection and then carefully removed. The incision was sutured and disinfected with povidone iodine. Three weeks post-injection, the efficiency of SLC7A11/YTHDC2 knockdown was evaluated using immunofluorescence staining and Western blot analysis.

### Plasmids

Lentiviral constructs targeting mouse SLC7A11 and YTHDC2 were produced following the pLKO.1-puro vector guidelines. The oligonucleotides for knocking down mouse SLC7A11 were 5'-CATTAGCAGTCCCGATCTTTG-3'. The oligonucleotides for knocking down mouse YTHDC2 were 5'-GATCATGCATCTCCTATATAA-3'.

To investigate how m^6^A interactions affect substrate selectivity and the *in vivo* function of YTHDC2, we constructed pRP (Exp)-EGFP/Puro-EF1A>FLAG/(mYTHDC2[NM_-_001163013] *SNP) (VectorBulider) plasmids to identify mutations in the YTHDC2 genes (W1375A and L1380A) (YTH- mut), thereby disrupting the m^6^A interactions.

### Quantitative analysis of m^6^A levels in mRNA

Quantitative analysis of m6A modifications in mRNA was performed using liquid chromatography-tandem mass spectrometry (LC-MS/MS). RNA was isolated from samples using RNAiso plus (Takara, Kyoto) according to the manufacturer's instructions. Subsequently, mRNA was enriched using the Dynabeads^TM^ mRNA Purification Kit (Invitrogen, Paisley, UK). For m^6^A quantification, 200 ng of polyadenylated mRNA was digested in 25 μL of ammonium acetate buffer (20 mM, pH = 5.3) containing 1.2 U of nuclease P1 (Sigma) at 42 °C for 2 h. Following digestion, 3 μL of ammonium bicarbonate (NH_4_HCO_3_) (1 M) and 1 U of alkaline phosphatase (Sigma) were added, and the mixture was incubated at 37 °C for an additional 2 h. The sample was then diluted to 50 μL with formic acid, filtered through a 0.22 μm syringe filter (Millipore), and 5 μL of the filtrate was injected into the LC-MS/MS.

Nucleosides were separated using a reversed-phase ultra-high performance liquid chromatography (UHPLC) system equipped with a C18 column. Mass spectrometric detection was performed on an Orbitrap Fusion Tribrid LC-MS system (Thermo, Waltham, MA, United States of America) in positive electrospray ionization mode. Quantification of nucleosides was based on retention times and transitions from nucleoside to base ions: adenosine (A) at m/z 268 -136 and m^6^A at m/z 282 -150. The concentrations of A and m^6^A in samples were calculated using a standard curve generated from reference standards run in the same batch. The m^6^A modification level was determined as the percentage of m^6^A relative to the total amount of A, with normalization for the amount of mRNA injected from different samples.

### RNA stability

Primary astrocytes were treated with actinomycin D at a final concentration of 5 μg/mL for specified durations before collection. Total RNA was extracted and analyzed using qRT-PCR. The mRNA half-life (t_.5_) was calculated using the formula: ln (2)/-slope, with normalization to GAPDH mRNA levels.

### Polysome profiling

For polysome profiling, a freshly prepared 5-50% linear sucrose gradient was generated in ultracentrifuge tubes (Beckman) using an automated gradient maker (BioComp). Cells were pre-treated with 100 μg/mL cycloheximide (CHX) (MCE) at 37 °C for 10 min, washed twice with ice-cold PBS containing 100 μg/mL CHX, and then collected. Cells were lysed in a hypertonic buffer containing 20 mM Tris-HCl (pH 7.4), 300 mM NaCl, 10 mM MgCl2, 0.5% sodium deoxycholate, 1% Triton X-100, 1 mM DTT, 100 μg/mL CHX, and 300 U/mL RNase inhibitor. Cell debris was removed by centrifugation at 16,000 × g for 10 min at 4 °C. Subsequently, 500 μL of the supernatant was layered onto the sucrose gradient and centrifuged at 4 °C and 36,000 rpm for 2 h (SW41Ti rotor, Beckman). Gradient fractionation and analysis of the samples were performed using a piston gradient fractionator (BioComp) and a fraction collector (Gilson, Villier Le Bel, France). To normalize, 5 ng of polyadenylated synthetic firefly luciferase mRNA (Promega, Southampton, UK) was added to each fraction. RNA was extracted from each fraction for qRT-PCR analysis.

### Dual-luciferase reporter assay

Fragments of SLC7A11 wild-type (-WT) or SLC7A11 mutant (-Mut) (where m^6^A was substituted with C) were inserted into the pRP (Exp)-Puro-EF1A>Luciferase: (mSLC7A11_3'UTR_1862-4861bp: SNP) (VectorBulider) plasmid to create SLC7A11-WT and SLC7A11-Mut plasmids. Seventy-two h post-transfection, luciferase activity was measured using the Dual-Luciferase Reporter Assay System (Promega). Relative firefly luciferase (Fluc)/Renilla luciferase (Rluc) activity was calculated by normalizing the activity of firefly luciferase to that of Renilla luciferase. Each experiment was conducted in triplicate for each group.

### RNA immunoprecipitation (RIP)

The RIP assay was performed using the Magna RIP Kit (Millipore) per the manufacturer's protocol. Cells grown on 15 cm plates were rinsed with ice-cold PBS and harvested in RIP lysis buffer supplemented with protease and RNase inhibitors. YTHDC2 antibodies (ab220160, Abcam) and nonspecific mouse IgG (Millipore) were attached to protein A/G magnetic beads through a 30-min incubation at room temperature. This was followed by three rinses and an overnight incubation with the cell lysate at 4 °C. Following six rinses, beads were treated with proteinase K at 55 °C for 30 min to digest proteins. RNA from both the input and the immunoprecipitated samples was then isolated using RNAiso plus, prepared for further analysis, and analyzed by either next-generation sequencing (NGS) or qRT-PCR.

### Methylated RNA immunoprecipitation sequencing (MeRIP-seq)

Total RNA was isolated from cellular samples using the RNAiso plus kit (Takara), according to the manufacturer's instructions. Subsequently, mRNA was enriched using the Dynabeads™ mRNA Purification Kit (Invitrogen). MeRIP-seq analysis was outsourced to Cloudseq Biotech Inc. (Shanghai, China). In summary, m^6^A RNA immunoprecipitation was conducted using the a GenSeq™ m6A RNA IP Kit (GenSeq) to prepare both input and m^6^A IP samples for next-generation sequencing. A sequencing library was generated using the NEBNext^®^ Ultra II Directional RNA Library Prep Kit (NEB), and its quality was assessed with the Agilent (Santa Clara, CA, United States of America) BioAnalyzer 2100 system. Sequencing of the library was carried out on an Illumina Hiseq platform, generating 150 bp paired-end reads.

### Extraction and culture of primary astrocyte cells

After euthanizing P1-2 mice, their brains were removed, and the temporal lobe tissue was collected. Under microscopic examination, the meninges were separated, and the temporal lobe tissues were finely minced using surgical scissors. This tissue was then subjected to enzymatic digestion in a 0.25% solution of pancreatic enzymes for 15 min to promote the release of cells. The digested material was filtered through a 100-μm sieve to obtain a suspension of astrocyte cells. The resulting suspension was cultured in Dulbecco's modified eagle medium (DMEM) enriched with 10% fetal bovine serum (FBS) and 1% penicillin/streptomycin. Cells were counted and seeded in T75 flasks at a density of 1 × 10^7^ cells per well. Following a 7-day culture period, allowing astrocytes to reach full growth and microglia to either become visible or detached, the flasks were vibrated at 180 rpm for 30 min on a shaker to separate the microglia, which were subsequently discarded with the supernatant. The culture received 2 mL of fresh medium and was again agitated at 240 rpm for 6 h to remove oligodendrocyte precursor cells (OPCs). After discarding the second supernatant and adding 2 mL of fresh medium, the prepared culture was set for further experimentation.

### Extraction and culture of primary neural cells

Primary neurons were isolated using neonatal mice aged 1-2 days. The mice were humanely euthanized, and their brains were extracted to retrieve temporal lobe tissue. Under a dissecting microscope, the meninges were carefully stripped away, and the temporal lobe tissue was finely chopped using ophthalmic scissors. These tissue pieces were then enzymatically digested with a 0.25% pancreatic enzyme solution for 15 min, facilitating the separation of individual cells. The digested tissue mixture was then strained through a 100-μm mesh to yield a suspension of neuronal cells.

This neuronal suspension was cultured in a Neurobasal medium (Gibco, USA), enriched with 10% FBS, 2% B27 supplement, 1% glutamine, and 1% penicillin/streptomycin. The cells were quantified and plated into six-well plates, maintaining a density of 5 × 10^5^ cells per well. After an incubation period of 7-10 days, the neuronal cells began exhibiting significant neurite outgrowth. Following this initial growth period, the culture medium was replaced with various types of conditioned media from astrocytes for further studies. Primary neurons were pre-treated with 100 μM pilocarpine or Kainic acid (KA) for 12 h, then co-cultured with primary astrocytes separated by a cell strainer for 48 h.

### Quantitative real-time polymerase chain reaction (RT-qPCR)

Analyses using RT-qPCR were performed on mouse hippocampal tissue and primary astrocytes to assess target gene expression levels. RNA was extracted from these cells using a column purification kit (Takara, Japan), per the manufacturer's instructions. This extracted RNA was then converted into cDNA through reverse transcription. The expression levels of specific genes in different astrocyte samples were measured using the TB Green method (Table S2). Quantification of gene expression was conducted using the 2^-ΔΔCT^ method, as outlined in the detailed protocol.

### Western blot

Mouse hippocampal tissues or primary astrocytes were homogenized in cold RIPA buffer (P101, Epizyme Biotech) supplemented with protease and phosphatase inhibitors (GRF101,102, Epizyme Biotech). The homogenates were incubated on ice for 30 min, followed by centrifugation at 15,000 *× g* for 20 min at 4 °C to remove cell debris. The BCA Protein Assay Kit (ZJ102, Epizyme Biotech) was utilized to determine protein concentrations. Lysates were diluted with 4× loading buffer (LT-101S, Epizyme Biotech) and boiled for 5 min at 95 °C before being separated on a 10% SDS-PAGE gel. Furthermore, proteins from the gel were transferred to a PVDF membrane, which was blocked with 5% non-fat milk in TBST buffer at room temperature for 60 min. The membrane was incubated overnight at 4 °C with primary antibodies diluted in 3% bovine serum albumin (BSA) on a shaker. After washing six times in TBST (10 min each), the membrane was incubated with secondary antibodies in 5% milk at room temperature on a shaker for 30 min, followed by three TBST washes. The secondary antibodies used were goat anti-rabbit IgG-HRP (LF102, Epizyme Biotech) and goat anti-mouse IgG-HRP (LF101, Epizyme Biotech). Protein visualization was achieved using ECL reagent (E412, Vazyme) and captured with a BioRad imaging system.

### Immunofluorescence

Mice were anesthetized and perfused with 4% paraformaldehyde. Their brains were then carefully removed and fixed in 4% paraformaldehyde at 4 °C overnight. To facilitate cryoprotection, brains were sequentially soaked in 20% sucrose solution at 4 °C overnight, followed by immersion in 30% sucrose solution at 4 °C for 72 h. The brain tissues were then embedded in an OCT compound and sectioned dorso-ventrally at 15 μm thickness using a cryostat (RWD Life Science, Shenzhen, China).

For immunofluorescence staining, tissue sections were thawed at room temperature for 15 min and rinsed thrice with PBS for 5 min per wash. To permeabilize the cell membrane and minimize background staining, sections were incubated in 0.3% Triton X-100 in PBS for 15 min. Subsequently, sections were blocked with 5% BSA for 30 min, then incubated with primary antibodies at 4 °C overnight. These antibodies targeted various markers including IBA1(sc-32725, Santa Cruz), xCT (ANT-111, Alomone), iNOS (ab178945, Abcam), GFAP (14-9892-82, Invitrogen), C3 (21337-1-AP, Proteintech), YTHDC2 (ab220160, Abcam), NRF2 (80593-1-RR, Proteintech), EAAT1 (20785-1-AP, Proteintech), EAAT2 (22515-1-AP, Proteintech), and OLIG2 (AB9610, Millipore).

After incubation with primary antibodies, sections were washed thrice with PBS to remove unbound antibodies and incubated with secondary antibodies, including Alexa Fluor 488-conjugated donkey anti-mouse (A32766, Invitrogen), Alexa Fluor 568-conjugated donkey anti-rabbit (A10042, Invitrogen), and Alexa Fluor 647-conjugated donkey anti-rabbit (A32795, Invitrogen) for 2 h at room temperature. Following secondary antibody incubation, sections were mounted using a DAPI-incorporated anti-fade mounting medium and observed under a fluorescence microscope (ZEISS, Axio Imager M2, Germany).

### Statistical analysis

Statistical analyses were performed using GraphPad Prism 9 (GraphPad Software, LLC, San Diego, CA, USA). All data are presented as mean ± standard error of the mean (SEM). Sample sizes (*n*) are indicated in the figure legends. The normality of data distribution was assumed but not formally tested. Comparisons between two groups were assessed using an unpaired Student's *t*-test. For multiple group comparisons, one-way analysis of variance (ANOVA) followed by Bonferroni's post-hoc test was applied. To evaluate results involving two independent variables, two-way ANOVA was performed. For repeated measures experiments, two-way repeated measures ANOVA was employed. For MRS data, we used two-sided paired Student's *t*-tests, while for small sample analyses, we used the Kruskal-Wallis H test. Experimental procedures were randomized to minimize the impact of confounding variables, including the selection of mice for experiments and the order of treatments. The experimenters were blinded to the group allocation of the mice. Imaging was conducted consistently across various conditions. For quantification, cells and fields of view from brain sections were randomly selected from designated areas of interest.

## Supplementary Material

Supplementary figures, table, and information.

## Abbreviations

AED: anti-epileptic drug
C3: complement component 3
CHX: cycloheximide
DMEM: Dulbecco's modified eagle medium
EAAT: excitatory amino acid transporter
EEG: electroencephalogram
EMG: electromyographic
FBS: fetal bovine serum
GABA: gamma-aminobutyric acid
IBA-1: ionized calcium-binding adaptor molecule
iNOS: inducible nitric oxide synthase
KA: kainic acid
MeRIP-qPCR: methylated RNA immunoprecipitation-qPCR
NGS: next-generation sequencing
NRF2: nuclear factor erythroid 2-related factor 2
OLIG2: oligodendrocyte transcription factor
OPC: oligodendrocyte precursor cell
qRT-PCR: quantitative real-time polymerase chain reaction
RIP-qPCR: RNA immunoprecipitation-qPCR
RMP: resting membrane potential
SEM: standard error of the mean
SRS: spontaneous recurrent seizures
TLE: temporal lobe epilepsy
TLE-HS: temporal lobe epilepsy with hippocampal sclerosis
UTR: untranslated region
xCT: SLC7A11
