# Supplementary material for: The m6A reader YTHDC2 promotes the pathophysiology of temporal lobe epilepsy by modulating SLC7A11-dependent glutamate dysregulation in astrocytes
Source: Theranostics. 2024 Sep 3;14(14):5551–70. doi: 10.7150/thno.100703 (PMC11413790; doi:10.7150/thno.100703)
Supplement: Supplementary file 1 — Supplementary figures, table, and information. [file thnov14p5551s1.pdf]

Supplementary materials

The m6A reader YTHDC2 promotes the pathophysiology of temporal lobe epilepsy by modulating SLC7A11-dependent glutamate dysregulation in astrocytes

Kai Zhang, Zhiquan Yang, Zhuanyi Yang, Liangchao Du, Yu Zhou, Shiyu Fu, Xiaoyue Wang, Xing Li, Dingyang Liu, Xinghui He*

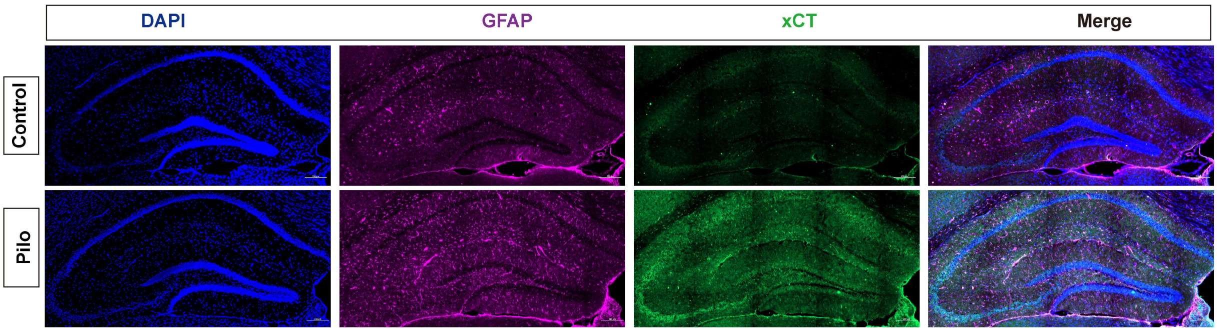

Figure S1. Representative immunofluorescence image of GFAP and xCT co-localization in the mouse hippocampus (scale bar = 200 μm)

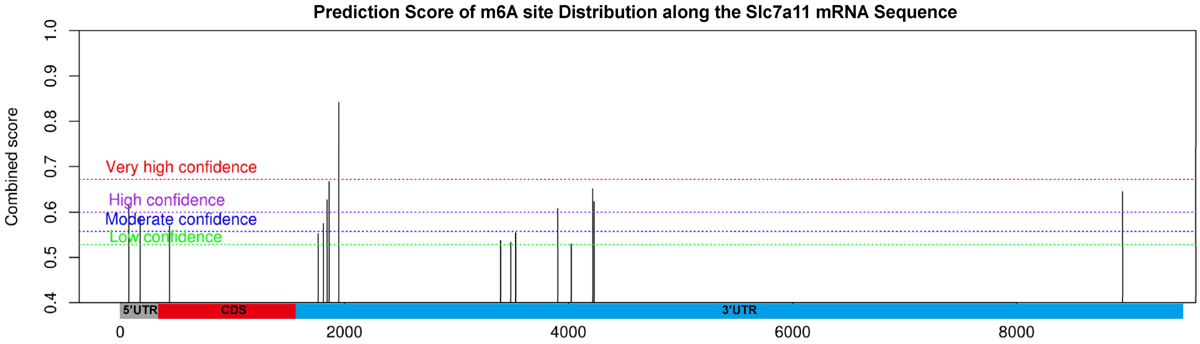

Figure S2. Predicted m6A sites in SLC7A11 mRNA identified by the SRAMP algorithm.

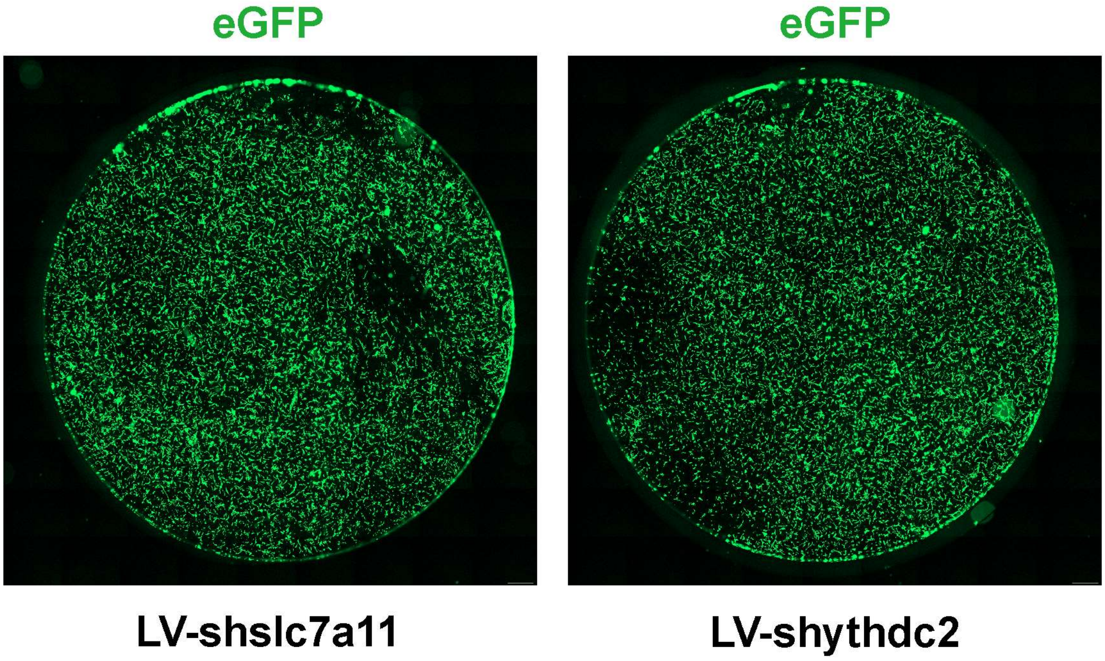

Figure S3. Representative immunofluorescence image of primary astrocytes transfected with

lentivirus.

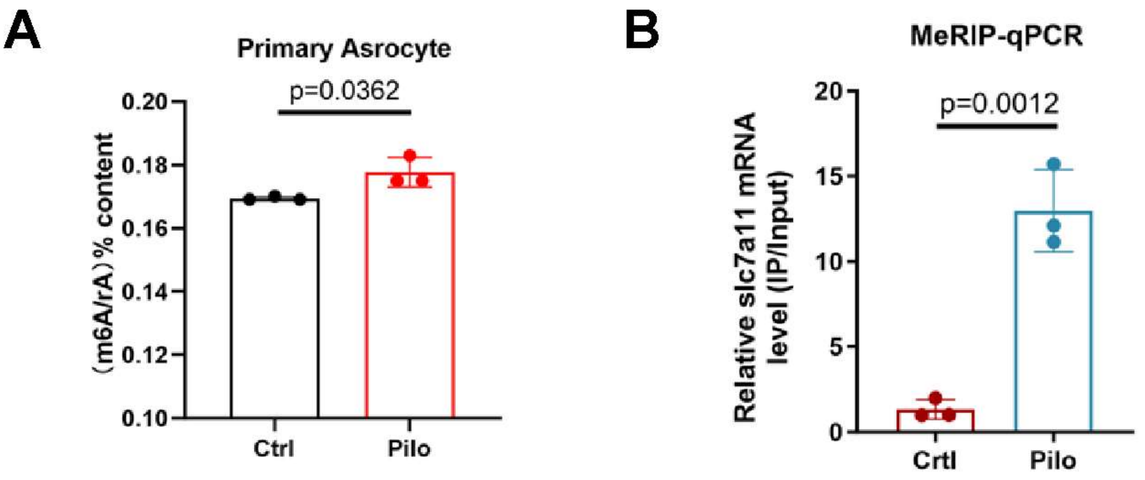

Figure S4. (**A**) Methylation modification of m6A in primary astrocytes after stimulation with pilocarpine and in the control group (*p = 0.0362, n = 3*). (**B**) MeRIP-qPCR analysis of hippocampal SLC7A11 m6A methylation modification in the pilocarpine and control groups (*p = 0.0012, n = 3*); two-sided unpaired Student's t-tests.

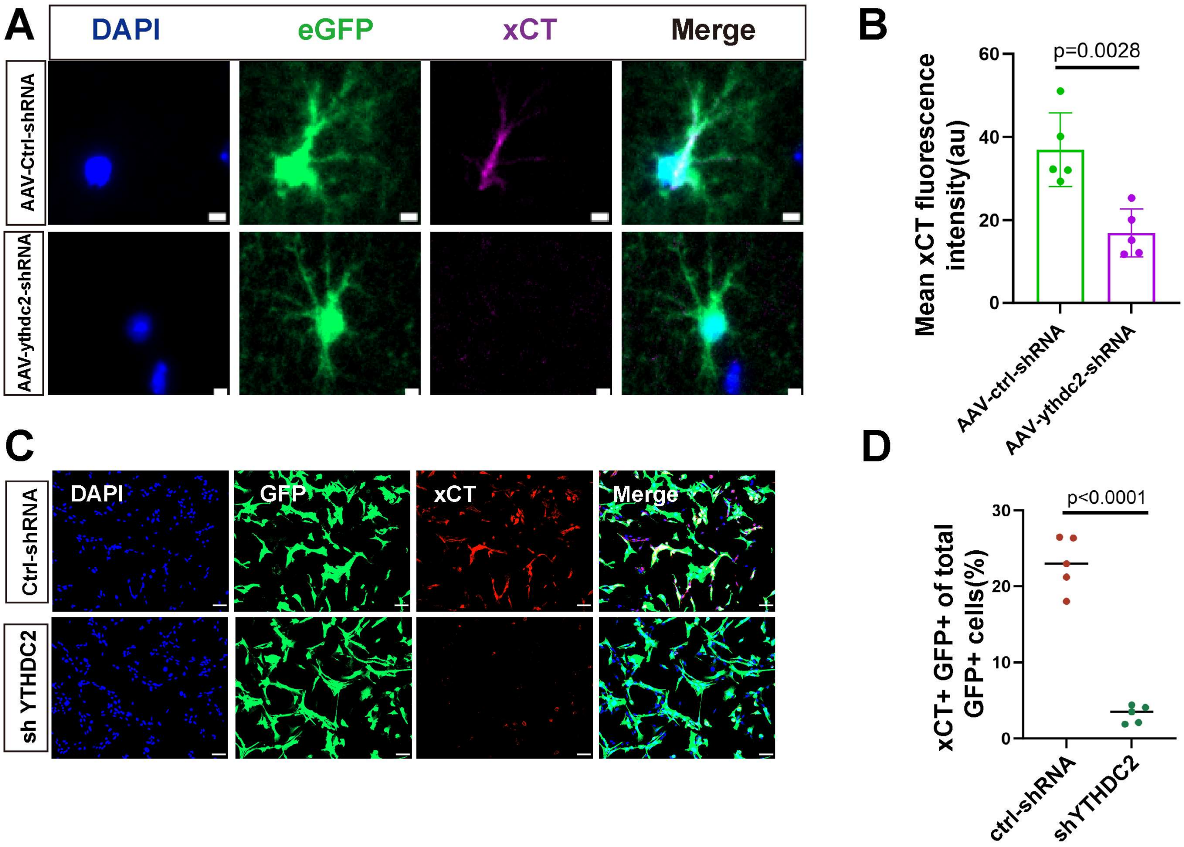

Figure S5. (**A, B**) Immunofluorescence staining of xCT protein fluorescence intensity in astrocytes of the YTHDC2-knockdown group and control groups (*p = 0.0028, n = 5*). scale bar = 2 μm. (**C, D**) Cell-immunofluorescence of xCT expression after pilocarpine stimulation in the YTHDC2-knockdown group and control groups (*p < 0.0001, n = 5*). scale bar = 50 μm. two-sided unpaired Student's *t*-tests (B, D).

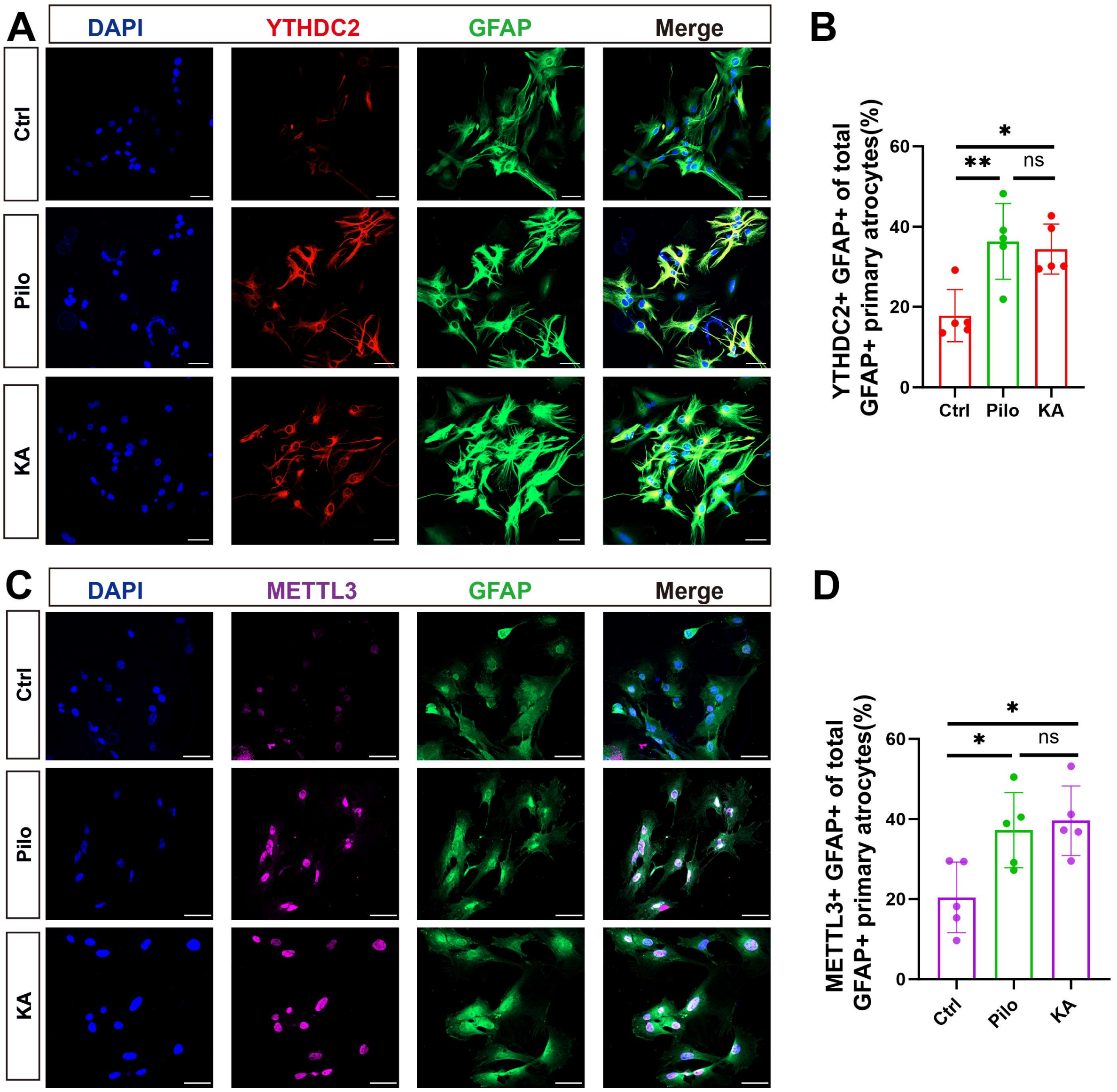

Figure S6 (A, B) Immunofluorescence staining of YTHDC2 in astrocytes treated with pilocarpine ($p = 0.0058, n = 5$) or KA ($p=0.0115, n = 5$). Scale bar = 50 μm. **(C, D)** Immunofluorescence staining of METTL3 in astrocytes treated with pilocarpine ($p = 0.0298, n = 5$) or KA ($p = 0.0141, n = 5$). scale bar = 50μm. one-way ANOVA with Bonferroni's post hoc test (B, D).

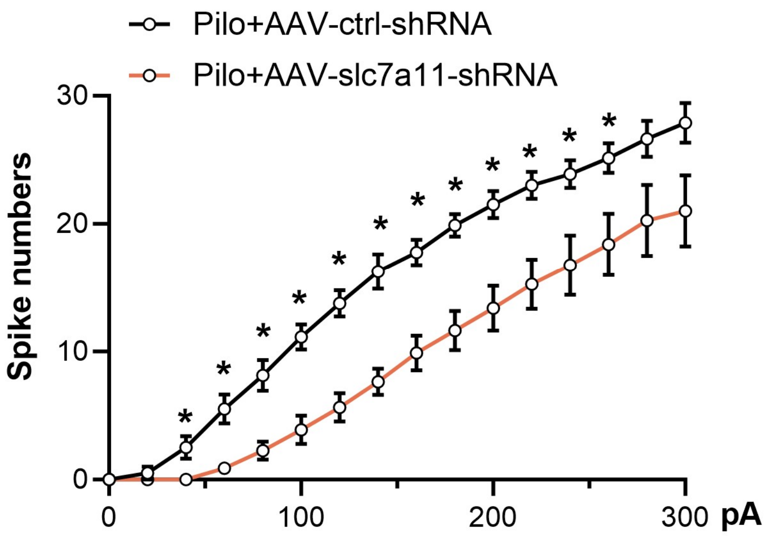

Figure S7 A range of current injections versus the number of spikes between AAV-ctrl-shRNA and AAV-slc7a11-shRNA group; two-side unpaired Student's *t*-test. * $p < 0.05$

Table S1 Human participant infromation

Patients	Sex	Age	Side	Histology	Experiment
TLE1	Male	23	Left	Hippocampal sclerosis	MRS
TLE2	Male	35	Left	Hippocampal sclerosis	MRS
TLE3	Female	29	Right	Hippocampal sclerosis	MRS
TLE4	Male	35	Left	Hippocampal sclerosis	MRS, IF
TLE5	Female	32	Right	Hippocampal sclerosis	IF
TLE6	Female	38	Left	Hippocampal sclerosis	IF
TLE7	Male	30	Right	Hippocampal sclerosis	IF
TLE8	Female	44	Left	Hippocampal sclerosis	IF
Normal1	Female	21	Right	-	IF
Normal2	Male	28	Left	-	IF
Normal3	Female	24	Right	-	IF
Normal4	Male	26	Right	-	IF
Normal5	Male	16	Left	-	IF

Table S2. Primer sequences used for qPCR

Gene	Forward Primer (5'-3')	Reverse Primer (5'-3')
Slc7a11	ATCTCCCCCAAGGGCATACT	GCATAGGACAGGGCTCCAAA
Actb	TATAAAACCCGGCGGCGCA	TCATCCATGGCGAACTGGTG
Ythdc2	ACGGTGACCAGAGAGAAATGG	GGTCATCATTGCATGAGCTGT
Ythdc1	CGGGAGGAGAAAGATGGGGA	TGTCGCTTGGTGTCAGTAGAC
Mettl3	CTTGCCATCTCTACGCCAGA	TCATGGCAGACAGCTTGGAG
Mettl4	AAGCGAGGGTTTGTTTCCGA	AAAGGTGAGTGTCCGTCAGG
Mettl14	CTTGGGAGAGTATGCTTGCG	CCTTTGATCCCCATCAGGCA
Alkbh5	GTGACTGTGCTCAGTGGGTA	TTCCAATCGCGGTGCATCTA
Fto	GGGACATCGAGACACCAGGA	AGGTGCCTGTTGAGCACTCT
Ythdf1	CCGTGATACACAGGAGGTGC	TCGATTCTGTCTTTCCTTACGCA
Ythdf2	CAGGCAAGGCCGAATAATGC	TCTCCGTTGCTCAGTTGTCC

Ythdf3	ACTAGCGTGGATCAGAGACCT	GGAAATCCAATGGACGGAGC
Nrf2	GCCCTCAGCATGATGGACTT	AACTTGTACCGCCTCGTCTG
Atf4	CCTATAAAGGCTTGCGGCCA	GATTTCGTGAAGAGCGCCAT
Bap1	TTGCAAACTATGAGGCCTGTCT	TTGGCCAGCATTCCTTCCTGA
Tp53	ATCCTGGCTGTAGGTAGCGA	CCATGGCAGTCATCCAGTCT

Knockdown sequences of adeno-associated virus (AAV)

1. GOSV0334324 MIR155(**Slc7a11**) *3 CV258: **GFAP**-EGFP-MIR155

CTGGAGGCTTGCTGAAGGCTGTATGCTGATAACCTGGAGACAGCGAACAGTTTTGGCCACTGACTGACTGTTCGCTCTCCAGGTTATCAGGACACAAGGCCTGTTACTAGCACTCACATGGAACAAATGGCCCGGGCCCTTTAAACTGGAGGCTTGCTGAAGGCTGTATGCTGAAATCAGTCCTGCGACTGCCAGTTTTGGCCACTGACTGACTGGCAGTCAGGACTGATTTCAGGACACAAGGCCTGTTACTAGCACTCACATGGAACAAATGGCCCTTAATTAACTGGAGGCTTGCTGAAGGCTGTATGCTGAATAGTGCCGGGATGAAGAGAGTTTTGGCCACTGACTGACTCTCTTCACCGGCACTATTCAGGACACAAGGCCTGTTACTAGCACTCACATGGAACAAATGGCCC

2. GOSV0334325 MIR155(**Ythdc2**) *3 CV258: **GFAP**-EGFP-MIR155

CTGGAGGCTTGCTGAAGGCTGTATGCTGTGCAATGGCATTCTCAAGAGTGTTTTGGCCACTGACTGACACTCTTGAATGCCATTGCACAGGACACAAGGCCTGTTACTAGCACTCACATGGAACAAATGGCCCGGGCCCTTTAAACTGGAGGCTTGCTGAAGGCTGTATGCTGTACAAAGCATAGCTGCCCGTTGTTTTGGCCACTGACTGACAACGGGCATATGCTTTGTACAGGACACAAGGCCTGTTACTAGCACTCACATGGAACAAATGGCCCTTAATTAACTGGAGGCTTGCTGAAGGCTGTATGCTGTAAACCTGCAGACTGTTCTTCGTTTTGGCCACTGACTGACGAAGAACACTGCAGGTTTACAGGACACAAGGCCTGTTACTAGCACTCACATGGAACAAATGGCCC
